# Organic-Dominated Nanozymes for Pesticide Detection: Toward Sustainable Agricultural Monitoring

**DOI:** 10.1021/acs.jafc.5c06721

**Published:** 2025-08-26

**Authors:** Eslam M. Hamed, Kustomo Kustomo, Mostafa M. ElSaady, Xiaoli Wu, Fun Man Fung, Sam F. Y. Li

**Affiliations:** † Department of Chemistry, Faculty of Science, 37580 National University of Singapore, 3 Science Drive 3, Singapore 117543, Singapore; ‡ Department of Chemistry, Faculty of Science, Ain Shams University, Abbassia, Cairo 11566, Egypt; § Department of Chemistry, Faculty of Science and Technology, Universitas Islam Negeri Walisongo Semarang, Jl. Prof. Hamka, Ngaliyan, Semarang, Central Java 50185, Indonesia; ∥ Institute of Medicinal Plant Development, 198148 Chinese Academy of Medical Sciences & Peking Union Medical College, Beijing 100193, China; ⊥ School of Chemistry, 8797 University College Dublin, Belfield, Dublin 4, D04 C1P1, Ireland; # UCD Geary Institute for Public Policy, University College Dublin, Belfield, Dublin D04 N9Y1, Ireland; ∇ Department of Pharmacology, Yong Loo Lin School of Medicine, National University of Singapore, Singapore 117600, Singapore

**Keywords:** nanozymes, sustainable agriculture, organic-dominated nanozymes, biodegradability, soil health, pesticides sensing

## Abstract

Nanozymes are synthetic enzymes that mimic natural enzymes with superior stability and cost-efficiency. Organic-dominated nanozymes overcome key limitations of inorganic variants, such as toxicity and environmental persistence, by offering biocompatible alternatives. Their applications in sustainable agriculture include pesticide sensing, nutrient management, and soil health monitoring. Advances in polymer-based, hybrid, and dendritic designs have enhanced catalytic specificity and scalability, though challenges remain in field performance and mass production. Future efforts will focus on multifunctional, stimuli-responsive nanozymes using green synthesis methods, promising transformative impacts on agricultural sustainability and food security. Additional potential lies in environmental remediation, postharvest preservation, and precision agriculture, enabling resilient crops and efficient resource use. However, long-term ecological effects, scalable synthesis, and regulatory frameworks require further study. Integrating emerging technologies could optimize smart fertilizers and crop protection strategies, fostering environmentally friendly food production. Addressing these challenges will unlock the full potential of organic-dominated nanozymes in advancing sustainable agriculture.

## Graphical abstract

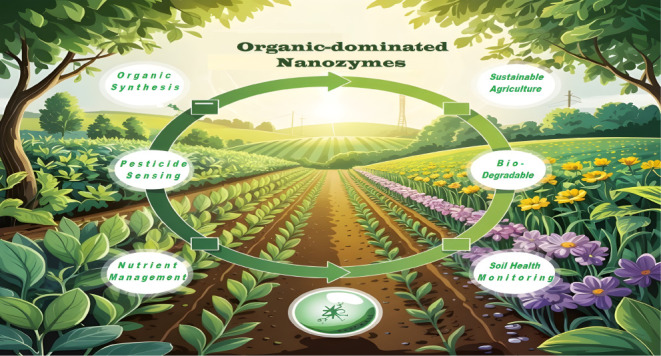

## 1 Introduction

Nanotechnology has witnessed remarkable advancements in recent years, with nanozymes emerging as a groundbreaking class of synthetic enzymes mimicking the catalytic activities of natural enzymes while offering enhanced stability, cost-effectiveness, and versatility.^1,2^ These nanomaterial-based catalysts have garnered considerable across various sectors, including biosensing,^3,4^ environmental remediation,^5–9^ and biomedical applications.^10,11^ Nanozymes are tiny materials and unique structures that imitate the function of natural enzymes and can perform similar chemical reactions. Most of these reactions involve redox processes, where electrons are transferred between molecules, influencing how effectively the nanozymes work. Being so small gives them a considerable surface area compared to their volume, which means they can interact with and change other substances more efficiently than natural enzymes.^12^ They can perform various enzyme-like activities, such as breaking down hydrogen peroxide or oxidizing other molecules.^13^ This versatility makes them useful in many different chemical reactions and environments. One of the most impressive things about nanozymes is their robustness. Unlike natural enzymes, which can be easily destroyed by heat or extreme conditions, nanozymes can keep working in harsh environments. This is because they are made of inorganic materials rather than proteins.^14^ As a result, nanozymes can function in very acidic or basic conditions, at high temperatures, and even in organic solvents.^15^ This makes them incredibly useful for industrial processes and environmental applications where natural enzymes would not survive.

The unique properties of nanozymes allow scientists to fine-tune them for specific uses. By adjusting their size, shape, or composition, researchers can optimize nanozymes for particular applications.^16^ Some nanozymes can even perform multiple enzyme-like functions at once, which is rare in nature. This multifunctionality opens up new possibilities for complex chemical reactions and integrated systems. As research in this field continues, scientists are working on making nanozymes even more specific and efficient. They are exploring ways to modify the surface of nanozymes, add other elements to them, or create hybrid structures to enhance their properties. The potential of nanozymes to solve problems in medicine, environmental science, and industrial processes makes them an exciting area of study across many scientific disciplines.^17–20^

Despite the extensive research on nanozymes, only a few studies have explored the classification of nanozymes based on their interaction mechanisms with one or more substrates or their cooperative designs for agricultural applications. To emphasize the significance of nanozymes in agriculture, this review focuses on tailored nanozyme systems designed to respond to specific or multiple substrates. Additionally, it examines recent agricultural applications of nanozymes, categorizing them into three main types: self-acting, synergistic, and remotely controlled systems. The review also highlights how nanozymes contribute to advancements in agriculture and outlines key design strategies to enhance their effectiveness in crop production, reduce plant diseases and pests, and promote sustainability. Lastly, we address the challenges and potential future directions in nanozyme development to fully harness their capabilities.

### 1.1 Traditional Nanozymes: Materials, Applications, and Limitations

Traditional nanozymes are primarily composed of inorganic materials, such as metal and metal oxide nanoparticles (e.g., iron oxide, ceria, and gold nanoparticles).^10,21–23^ These materials exhibit exceptional enzyme-like activities, including peroxidase (POD),^24^ oxidase (OXD),^3^ catalase (CAT),^25^ laccase (LAC),^26^ and superoxide dismutase (SOD)^27^ mimics. For instance, iron oxide nanoparticles display remarkable POD-like activity, catalyzing the oxidation of various substrates in the presence of hydrogen peroxide.^28^ Nanozymes have been extensively used in colorimetric and electrochemical sensing platforms to detect various analytes, including glucose, pesticides, and heavy metals.^29^ The catalytic properties of nanozymes have been harnessed for the degradation of pollutants and the removal of contaminants from water and soil.^30^ Nanozymes have also shown promise in cancer therapy, antimicrobial treatments, and tissue engineering due to their ability to modulate reactive oxygen species (ROS) levels and catalyze specific reactions in biological environments.^10,31–34^

In the biomedical field, nanozymes have opened new avenues for therapeutic interventions and diagnostic techniques. Their application in targeted drug delivery, cancer therapy, and managing oxidative stress-related disorders has garnered considerable attention.^35^ The multifunctional nature of nanozymes allows for the simultaneous performance of therapeutic and diagnostic roles, paving the way for integrated approaches in personalized medicine. Recent advancements include the development of nanozymes with multiple catalytic activities, such as those combining POD-like and CAT-like functions. These multifunctional nanozymes offer enhanced therapeutic potential, particularly in the treatment of diseases associated with oxidative stress.^35^

The multifaceted nature of nanozymes has led to their integration into numerous scientific and technological domains. In biosensing, these artificial catalysts have revolutionized the development of highly sensitive and specific detection methods for a variety of analytes.^36^ Their signal amplification properties and stability have facilitated the creation of robust sensors for point-of-care diagnostics and environmental monitoring. Environmental remediation represents another frontier where nanozymes have made substantial progress. Their capacity to catalyze the breakdown of persistent organic pollutants (POPs) and detoxify heavy metals positions them as valuable tools in water and soil treatment strategies.^37^ For example, iron oxide-based nanozymes have shown remarkable efficiency in degrading organic dyes and phenolic compounds in wastewater.^38^ The eco-friendly nature of many nanozymes and their reusability align well with the principles of green chemistry and sustainable environmental management.^39,40^

However, despite their numerous advantages, traditional nanozymes face several limitations. One primary concern is their potential toxicity, mainly inorganic nanozymes containing heavy metals, which may threaten living organisms and ecosystems.^41,42^ The persistence of these inorganic materials in the environment could result in long-term ecological consequences due to their limited biodegradability.^40^ Furthermore, many traditional nanozymes are associated with complex, energy-intensive synthesis processes involving harsh chemicals.^30,43^ Moreover, many nanozymes exhibit lower substrate specificity compared to natural enzymes, limiting their utility in specific applications.^44^

### 1.2 Advantages of Organic-Dominated Nanozymes

Artificial enzymes have been recently developed to overcome the limitations of natural enzymes. Although natural enzymes play quintessential functions in living organisms, their application in nonphysiological mediums is very limited because the production in large amounts is very difficult, and its catalytic activity is negatively affected by the external environment^45^ like high temperature, different pH, and ionic strength which can lead to destroying the protein and the function deactivation.^46^ Moreover, the expensive preparation, purification, and special storage conditions restrict their practical applications. Therefore, researchers pay more attention to developing highly stable enzymes in high amounts and lower costs. Based on stable catalytic activity, enzymes, especially organometallic and organic-dominated nanozymes, are considered more stable than natural enzymes.^47^ This provides the ability for a wide range of catalytic applications, including therapeutic drugs, filtering, imagining, sensing, and detection.^28^ Although both organic and inorganic nanozymes have been utilized in different potential applications, organic-based nanozymes surpass in four major disciplines: fabrication streamline, environmental eco-friendly, cost-effectiveness, and sustainability (Figure 1A). This section will discuss the advantages of organic enzyme production in more detail.

**Figure 1:**
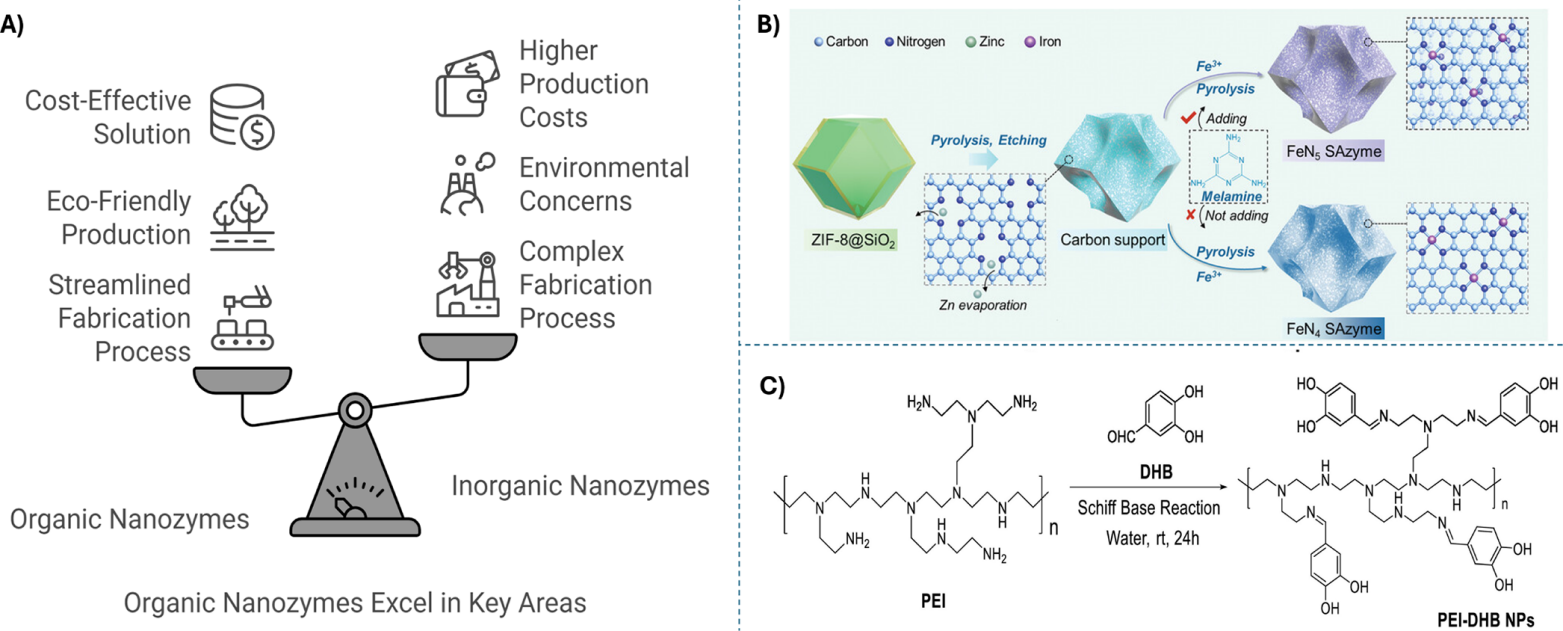
A) A brief comparison between organic and inorganic nanozymes. B) The synthesis process of bioinspired Fe-SAN as an example for inorganic nanozymes. Reprinted with permission from ref. 52. Copyright (2024) Wiley. C) The synthesis of PEI-DHB NPs by the Schiff base reaction as an example for organic-dominated nanozymes. Reprinted with permission from ref. 51. Copyright (2019) Elsevier.

In this review, we adopt the term “organic-dominated nanozymes” to refer to nanozymes in which organic components, such as peptides or supramolecular assemblies, play the dominant structural and functional role in catalysis. This term is chosen deliberately to distinguish such systems from conventional inorganic nanozymes or those with carbon-based but catalytically inorganic mechanisms.

#### 1.2.1 Fabrication Streamline

In contrast to the complex and the multistep treating processes for the inorganic nanozymes fabrication, organic-dominated nanozymes are produced more simply and effectively. In other words, the inorganic nanozymes fabrication process involves a long-term heating process and additional modification steps that can negatively affect the product yield and limit the mass-scale manufacturing for real-life applications.^48,49^ For example, Single-atom nanozymes (SANs) fabrication processes contain annealing at high temperatures for a long time and specific temperature rising rate, pyrolysis under the flow of inert gases, calcination to evaporate unwanted elements, doping of hetero elements to improve the catalytic activity and selectivity and/or organic solvent treatment to obtain active sites or adapting the internal structure (Figure 1B).^50^ in contrast to traditional nanozymes, organic-dominated nanozymes synthesis is faster, simpler, and more effective in large-scale production; their synthesis can easily be performed by mixing the precursors at room temperature over few hours without complex high-temperature regulation (Figure 1C). For instance, Yin et al. have developed a rapid and straightforward preparation process for a metal-free polymer nanozyme with POD-like activity. This organic nanozyme is prepared by mixing a water solution of Hyperbranched polyethylenimine (PEI) with dihydroxy benzaldehyde (DHB) at room temperature that are covalently bonded, forming PEI-DHB nanozyme.^51^ Therefore, it can be said that organic-dominated nanozymes can be fabricated in a much simpler route than inorganic nanozymes, which provides advantages of mass production and field application.

#### 1.2.2 Eco-Friendly, Nontoxic and Biocompatible

Compared with inorganic nanozymes, organic-dominated nanozymes have lower toxicity and are more biocompatible. There are only a few metal elements known as biocompatible elements, like iron and copper, because of their relatively high median lethal dose (LD_50_ > 5000 mg/kg), so they are categorized as “considerably biocompatible”. At the same time, their toxicity is different according to the compounds containing them. This means that most inorganic nanozymes threaten the environment and humanity because of the high toxicity levels of metals. Considering that the inorganic nanozymes draw a low risk despite their high toxicity in some applications that are away from direct interaction with living organisms or the environment, like ex-vivo biosensing or diagnostic assays, in case they are well contained after use. Meanwhile, implementing metallic nanozymes in in vivo sensing, pharmaceutical, cell-based assays, and agricultural applications poses a high risk because of their high toxicity.^46^ In contrast, organic-dominated nanozymes, even those that contain copper or iron as active metallic centers, are more biocompatible. For that, they are safer to be used in agricultural materials and living organisms (Figure 2A).^53–55^ For example, Dong et al. developed an amino acid–based organic nanozyme with POD-like activity that was employed for the detection of bio allergic molecules like histamine. After performing conventional cell viability assay incubation for 24 h, the developed organic nanozyme was verified to exhibit minimal toxicity and high biocompatibility, which supports its ability to be further used in different biological applications. Hence, organic-dominated nanozymes are considered alternative solutions for the inorganic ones in bio and environmental applications due to their lower toxicity and higher biocompatibility.

**Figure 2:**
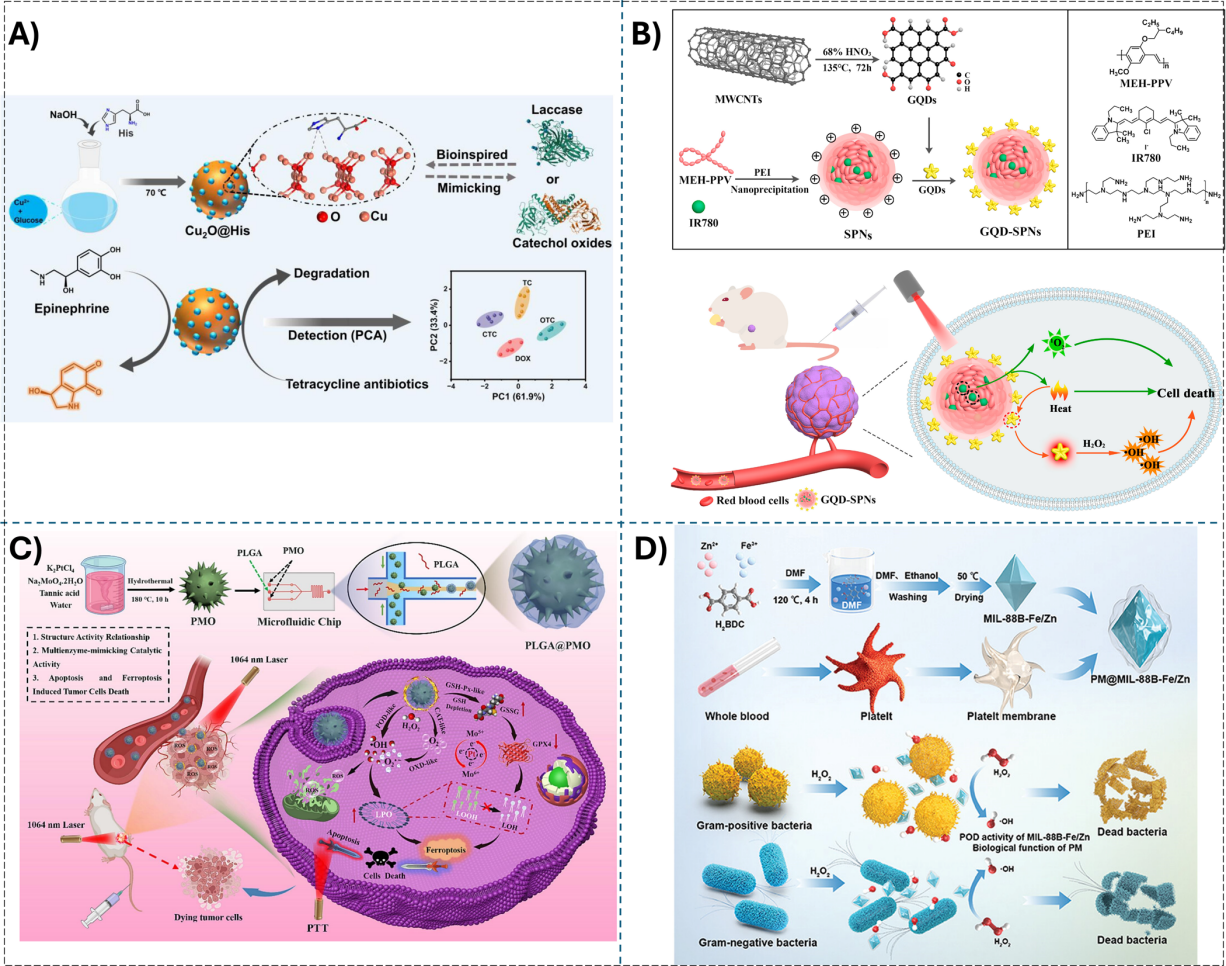
A) Synthesis and application of eco-friendly ultrasmall Cu_2_O@His with laccase- and catechol oxidase-like activity. Reprinted with permission from ref. 53. Copyright (2024) Elsevier. B) The preparation of scalable GQD-SPNs and underlying synergism of photothermal effect enhancing GQD-SPNs/nanozyme-mediated cancer catalytic therapy. Reprinted with permission from ref. 57. Copyright (2021) Elsevier. C) The synthetic procedures of PLGA@PMO and the therapeutic mechanism with dual-channel death strategies concerning apoptosis and ferroptosis. Reprinted with permission from ref. 59. Copyright (2024) Elsevier. D) Preparation scheme of PM@MIL-88B–Fe/Zn. Scheme of the biodegradable nanozyme for high-efficiency antibacterial therapy. Reprinted with permission from ref. 61. Copyright (2023) Wiley.

#### 1.2.3 Cost-Effectiveness and Scalability

Following from the discovery of nanoparticles mimicking enzyme-like effects, scholars kept developing nanozymes that ensure higher stability and lower cost than natural enzymes.^56^ Although inorganic nanozymes utilize noble metals like gold and platinum to enhance catalytic activity and stability, the price of these expensive meals (e.g., AuCl_3_ ∼$300/g) along with the multistep fabrication processes and thermal regulation treatments maximize the final product cost. On the other hand, organic nanozyme fabrication consumes cost-effective organic materials like polymers, small organic molecules, and biological materials. Metallic ions or metallic NPs like iron and copper are also used to create coordinate bonds with organic ligands in organic nanozyme, generating active sites with effective enzyme-mimicking catalytic reaction (Figure 2B).^55,57^ However, the prices of the transition metals used are still relatively lower than those of noble metals. The short-time fabrication advantage and the commercially available organic material and metallic ions with low prices (e.g., Urea ∼$0.2/g, and FeCl_2_·4H_2_O ∼$0.5/g) open access for scalability and mass production in an economic value. Additionally, some small molecules were also found to exhibit enzyme-mimicking activity. For example, Dichlorofluorescein was found to possess a POD-like activity and was used for easy and simple detection of H_2_O_2_ and glucose.^58^

#### 1.2.4 Sustainability and Biodegradability

Nanozymes have been utilized in environmental applications like environmental pollutant detection and degradation.^38^ On the other hand, while using inorganic nanozymes as a substrate for treating the environment, it is paramount not to cause secondary pollutants by leaching of metal ions and nondegradable substances. The difficulty of natural degradation of inorganic nanozymes hinders their wide usage in environmental applications. Addressing the pollution problems caused by nano and microscaled substances is a hot research area, and scholars are focusing on treating or developing sustainable and naturally degradable materials (Figure 2C&D).^59–61^ Hence, advancement in organic-based nanozymes enhances their natural degradation properties and sustainability. For example, Lee and Kamruzzaman introduced an entirely polymer and monomer-based nanozyme with POD-like mimicking activity.^40^ These organic base nanozymes witness a high biodegradability as they can damage their scaffold within 24h incubation at moderate temperature. These biodegradability properties solve the after-use pollution issue and broaden their applications in environmental and biological applications. Generally, organic-dominated nanozymes provide sustainable solutions for the challenges combatted by the inorganic ones and offer safe alternative and sustainable solutions.

The unique advantages of organic-dominated nanozymes, including streamlined fabrication, biocompatibility, cost-effectiveness, and biodegradability, position them as transformative tools for sustainable agriculture. Leveraging these properties, researchers have pioneered diverse applications that address critical agricultural challenges, from precision pesticide detection to soil health enhancement. The following sections explore how these intrinsic advantages translate into functional innovations, offering scalable solutions to reduce environmental contamination while improving crop productivity.

### 1.3 Organic-Dominated Nanozymes and Their Potential in Agriculture

Organic material-based nanozymes, referred to as organic-dominated nanozymes, employ organic substances as the core material to create nanozymes that integrate a minor metal catalyst, metal ion, or other catalytic agents, representing a novel category of nanozymes. Organic-dominated nanozymes are artificial enzyme-like materials composed predominantly of carbon-rich organic frameworks (e.g., polymers, covalent organic frameworks (COFs), metal–organic frameworks (MOFs) with organic ligands), with or without embedded metal ions. We define them as those derived from biodegradable organic building blocks, often prepared under mild conditions, with inherent biocompatibility and degradability, distinct from purely inorganic or metal oxide-based nanozymes. The components of organic-dominated nanozymes are varied, comprising naturally derived materials and synthesized biomaterials, including polymers, biomacromolecules, and associated organic chemicals.

On the other side, some SANs are structurally supported on carbonaceous or g-C_3_N_4_-based matrices and may appear similar to those classified under organic-dominated nanozymes. However, they are not included within our definition of organic-dominated nanozymes in this review. This distinction is made because SANs typically rely heavily on atomically dispersed transition metals (e.g., Fe, Co, Ni) as the primary active sites. These mechanistic features align more closely with inorganic catalysis paradigms than with biocompatible, green-synthesis, or metal-free approaches that characterize organic-dominated nanozymes. Therefore, while the support material may be organic in nature, the functional identity and synthetic origins of SANs justify treating them as a separate nanozyme subclass.

Recent progress in developing organic-dominated nanozymes, such as those based on polymer–monomer systems, has revealed their ability to undergo structural deformation and degradation under mild conditions.^40^ These degradable and biodegradable characteristics help mitigate pollution risks associated with structural breakdown and element leaching postusage, offering a more sustainable approach for nanozyme applications.^40^ Ultimately, the limitations associated with inorganic nanozymes underscore the need for alternative solutions, with organic-dominated nanozymes presenting a promising pathway toward sustainability.^62–67^

Organic-dominated nanozymes represent a promising alternative to traditional inorganic nanozymes, addressing many of the above-mentioned limitations. These novel catalysts primarily comprise organic compounds and frequently incorporate biomolecules or bioinspired structures to achieve enzyme-like activities.^68^ Organic-dominated nanozymes offer several advantages, particularly in sustainable agriculture, including biocompatibility, biodegradability, tunability, and environmentally friendly production (Figure 3A).^21^

**Figure 3:**
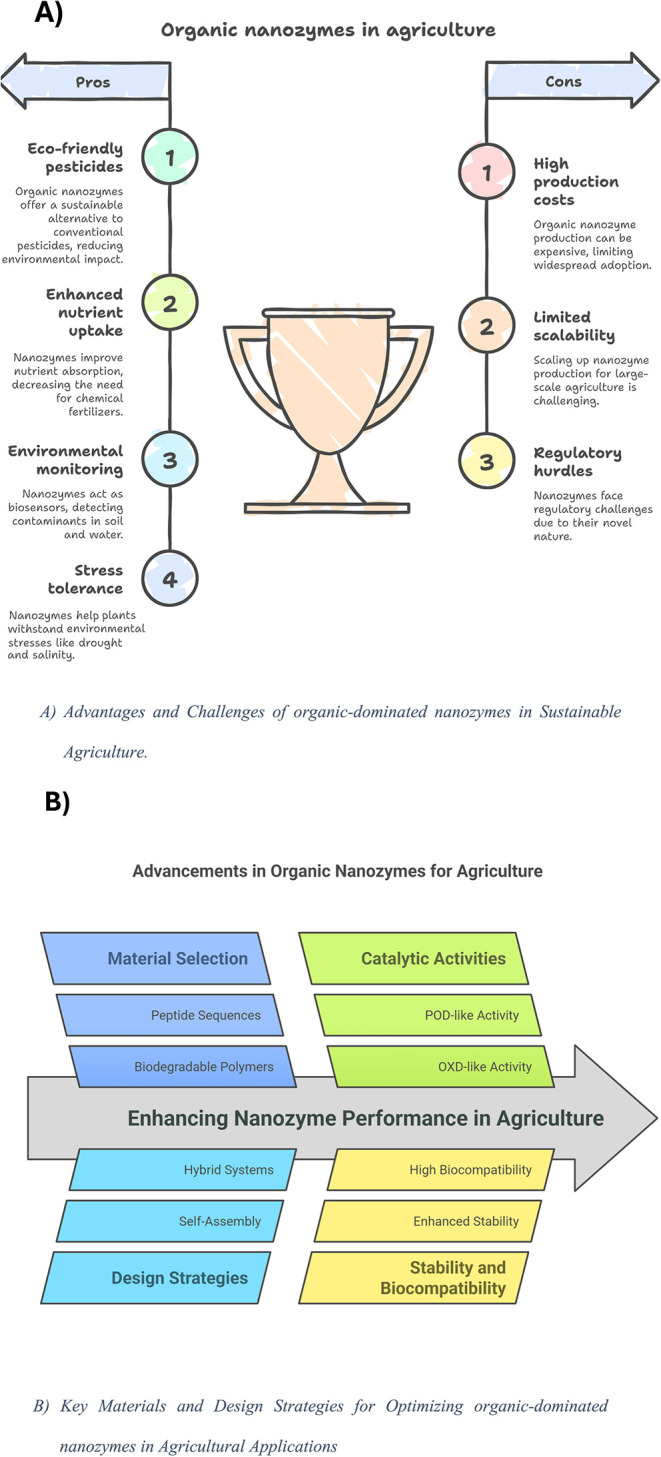
A) Advantages and challenges of organic-dominated nanozymes in sustainable agriculture. B) Key materials and design strategies for optimizing organic-dominated nanozymes in agricultural applications.

Organic-dominated nanozymes, like their inorganic counterparts, also mimic the activity of natural enzymes. They are made by combining metal ions or particles with organic materials, which helps create the structure of the particles and generates active sites for catalysis.^67^ For example, nanozymes derived from organic compounds are formed through particle development facilitated by interactions between Fe ions and nitrogen or oxygen atoms.^69^ These Fe–N and Fe–O coordination sites emulate the heme cofactor of hydrogen peroxidase to some extent, granting them POD-like activity. Consequently, the catalytic efficiency of these nanozymes depends on the coordination between metal ions and organic compounds (such as their functional groups). At the same time the fabrication method can affect the relationship between the metal ion’s resting state and its ability to exhibit specific catalytic activities, such as POD or OXD.

These nanozymes tend to be more compatible with living organisms, reducing concerns about toxicity to plants, beneficial microorganisms, and other nontarget organisms. Unlike their inorganic counterparts, organic-dominated nanozymes are designed to naturally degrade in the environment, thereby minimizing long-term ecological impacts.^2,44^ Their organic composition also provides greater flexibility in design, allowing for targeted modifications to suit specific agricultural applications. Furthermore, many organic-dominated nanozymes can be synthesized using green chemistry principles, reducing the environmental footprint of their production.^70^

In sustainable agriculture, organic-dominated nanozymes have the potential for various applications, such as crop protection, nutrient management, environmental monitoring, and/or stress tolerance. organic-dominated nanozymes could be eco-friendly alternatives to conventional pesticides, leveraging their catalytic activities to combat plant pathogens or degrade harmful chemicals.^71^ Additionally, organic-dominated nanozymes could enhance crops nutrient uptake and utilization, potentially reducing reliance on chemical fertilizers and improving soil health. As sensitive and specific biosensors, organic-dominated nanozymes could also detect pesticide residues, heavy metals, and other contaminants in soil and water.^7,71,72^ By modulating ROS levels or catalyzing the production of beneficial compounds, organic-dominated nanozymes could help plants mitigate various environmental stresses, such as drought or salinity.^73^

The field of organic-dominated nanozymes has seen significant progress in recent years, with researchers exploring various materials and design strategies. Researchers have developed enzyme-mimicking nanoparticles using biodegradable polymers, such as polydopamine (PDA) and poly­(lactic-*co*-glycolic acid) (PLGA), demonstrating POD-like and OXD-like activities.^74–78^ Additionally, short peptide sequences have been designed to self-assemble into nanostructures exhibiting catalytic properties, offering high specificity and biocompatibility of peptide-based nanozymes.^32,79^ DNA-based nanozymes, including G-quadruplexes and DNAzymes, have also shown promise as organic-dominated nanozymes with tunable catalytic activities.^4,80^ Furthermore, hybrid systems incorporating natural enzymes or other biomolecules onto organic nanoparticles have been developed to enhance stability and catalytic performance.^66,81,82^

More recently, nanozymes have shown significant potential in sustainable agriculture, offering promising solutions to longstanding challenges in crop protection, nutrient management, and environmental monitoring. Several innovative approaches warrant further investigation to further advance the field of organic-dominated nanozymes in sustainable agriculture. For example, developing nanozymes that can be activated or deactivated in response to specific environmental cues (e.g., pH, temperature, or light) could enable precise control over their activity in agricultural environments.^7,68,83^ Moreover, integrating multiple catalytic functions or combining sensing and catalytic capabilities into a single nanozyme could provide versatile tools for crop management and environmental monitoring.^6^

Inspiration could also be drawn from natural plant defense mechanisms or beneficial soil microorganisms to develop organic-dominated nanozymes that mimic these processes within agroecosystems. Additionally, embedding organic-dominated nanozymes into biodegradable matrices or combining them with beneficial microorganisms (nanobiocomposite formulation) could enhance their stability, delivery, and overall efficacy in agricultural applications. Furthermore, exploring methods for the controlled formation of organic-dominated nanozymes directly in plant tissues or soil environments could overcome challenges related to delivery and distribution (Figure 3B). By pursuing these innovative pathways, researchers can unlock the full potential of organic-dominated nanozymes in sustainable agriculture, contributing significantly to addressing global challenges in food security and environmental sustainability.^84^

The fields of agriculture, food, and environmental science are rapidly becoming significant focus areas for the application of nanozymes. Among these, nanozyme-based sensors face fewer challenges than other uses, as the detection methods for agricultural and food molecules share similarities with those employed in biomedical sensing. However, most nanozymes currently used in these applications are inorganic, making them less suitable for long-term use due to previously reported limitations.^41,85,86^

To better serve these fields, particularly in sensor technologies, there is growing interest in organic compound-based nanozymes. These organic-dominated nanozymes, which are expected to replace inorganic counterparts, offer advantages such as sustainability and cost efficiency. Nanozymes also exhibit antibacterial properties by generating ROS, which helps destroy bacterial films by killing the bacteria.^21^ This functionality can be utilized in food preservation to extend the shelf life of perishable goods by preventing bacterial contamination.^17^

For example, a nanozyme derived from biological materials, specifically bovine serum albumin (BSA) chelated with copper ions, demonstrated SOD-like catalytic activity. This nanozyme effectively enhanced bacteria-killing activity in vitro and significantly prolonged the storage life of fruits like apples and figs.^87^ In the agricultural and environmental sectors, nanozymes also hold the potential for promoting plant growth and enhancing crop yields. For instance, nanozyme-based nanofertilizers represent a promising application. However, current examples of nanozyme use in nanofertilizers remain limited and predominantly focus on inorganic nanozymes.^88–91^

## 2 Types of Organic-Dominated Nanozymes

Organic-dominated nanozymes, composed predominantly of carbon-based or organic macromolecular frameworks, represent an expanding class of enzyme mimics with diverse structural architectures and catalytic functions. Unlike their inorganic counterparts (e.g., metal oxides or single-atom catalysts), organic-dominated nanozymes offer biocompatibility, tunability, and structural diversity, making them promising for biomedical, environmental, and sensing applications (Table 1).^92^ This section categorizes the main types of organic-dominated nanozymes based on their material origin and structural features, including carbon-based nanomaterials, MOFs, COFs, and polymeric nanozymes.

**Table 1:** Comparative Analysis of Organic and Inorganic Nanozymes for Agricultural Applications

Aspect	Organic-dominated Nanozymes	Inorganic Nanozymes
**Synthesis Cost**	Low; uses cost-effective biomaterials (e.g., urea), and simple processes	High; relies on expensive noble metals (e.g., AuCl_3_) and complex energy-intensive processes
**Toxicity**	Low; biocompatible, minimal toxicity to plants and soil microbiota (e.g., amino acid–based nanozymes)	High; heavy metals (e.g., Cd, Pb) pose risks to ecosystems and human health
**Scalability**	High; one-pot, aqueous, or biocatalytic methods enable mass production with minimal equipment	Moderate; multistep, high-temperature processes (e.g., annealing, pyrolysis) limit large-scale production
**Biodegradability**	High; naturally degradable within hours to days, reducing environmental persistence	Low; persistent in environment, causing potential long-term ecological harm
**Environmental Impact**	Low; aligns with green chemistry, minimal waste from solvent-free or aqueous synthesis	High; hazardous chemicals and energy-intensive synthesis increase ecological footprint
**Catalytic Specificity**	Moderate; tunable but less precise than natural enzymes, improving with design advancements	Lower; often less specific due to broad reactivity of metal-based active sites

### 2.1 Metal-Free Organic-Dominated Nanozymes

Metal-free organic-dominated nanozymes are composed entirely of organic elements (typically carbon, hydrogen, nitrogen, and oxygen) without any added transition metals. Their catalytic activity originates from structural features such as conjugated π-systems, surface defects, or heteroatom doping (e.g., N, S). Examples include carbon-based materials like graphene oxide (GO), carbon dots (CDs), and graphitic carbon nitride (g-C_3_N_4_). Polymeric materials or molecularly imprinted polymers (MIPs) can also be classified as organic-dominated nanozymes if entirely composed of polymers without metal ions. These nanozymes are often preferred for biological and agricultural applications due to their high biocompatibility, low toxicity, and eco-friendly synthesis processes.

#### 2.1.1 Carbon-Based Organic-Dominated Nanozymes

Carbon-based nanomaterials such as graphene, CNTs, CDs, and fullerene derivatives are among the most widely studied organic-dominated nanozymes due to their excellent electrical conductivity, stability, and large surface area. Their intrinsic enzyme-like activities properties stem from surface defects, oxygen-containing groups, and dopants (e.g., N, S, or B).^93^

GO and its derivatives, for instance, have shown POD-like activity through electron transfer reactions with substrates like 3,3′,5,5′-tetramethylbenzidine (TMB) in the presence of H_2_O_2_. Catalytic behavior is often attributed to π-conjugated systems and the abundance of edge-plane defects which serve as active sites.

CDs and g-C_3_N_4_ are other notable carbon-based nanozymes. Doped CDs with nitrogen or metals exhibit significant enzyme-mimicking abilities,^94^ while g-C_3_N_4_, a polymeric material formed from triazine units, can mimic photoactivated OXD-like activity under visible light, useful in photocatalytic antibacterial and biosensing platforms (Figure 4A).^95^

**Figure 4:**
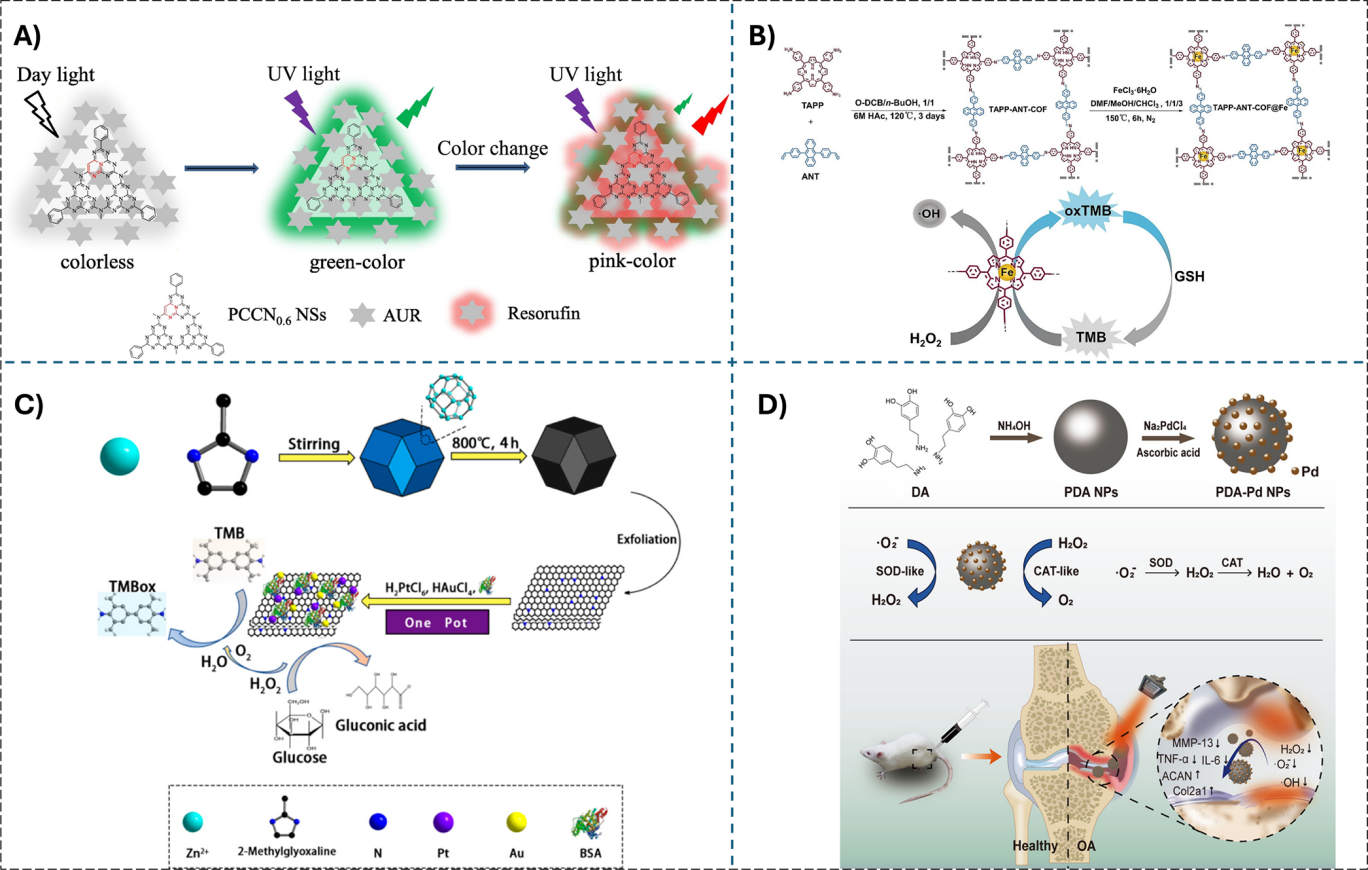
A) Color change mechanism of confidential information written by the mixture of phenyl- and carbon-modified g-C_3_N_4_ nanosheets and Amplex UltraRed as the fluorescent ink. Reprinted with permission from ref. 95. Copyright (2022) Elsevier. B) Illustration of the production pathway for TAPP-ANT-COF and TAPP-ANT-COF@Fe and the application in colorimetric GSH assay. Reprinted with permission from ref. 105. Copyright (2025) Elsevier. C) Schematic illustration of synthetic and catalytic procedure of BSA-PtAu@CNS nanozyme cascade bioplatform. Reprinted with permission from ref. 106. Copyright (2020) American Chemical Society. D) The synthesis process of PDA-Pd nanoparticles with CAT- and SOD- like activities and their anti-inflammatory capacity in vitro and in vivo. Reprinted from ref. 98.

Fullerenes and its derivatives are often nanoparticles that resemble SOD. The antioxidant properties of fullerenes and carboxyfullerenes in scavenging free radicals to protect brain cells were originally documented by Dugan et al. in the 19th.^96^ Qin and colleagues examined the ability of three polyhydroxylated fullerenes (fullerol) to chelate ferrous ions through its unique carbon cage structure and inhibits ferroptosis in nucleus pulposus cells.^97^ They established the Rap1/Keap1/Nrf2 signaling axis in regulating ferroptosis and showed that fullerol could eliminate ROS accumulation as a mitochondrial redox modulator, which underlies the antiferroptotic effects of fullerol. This established a strong basis for the clinical use of fullerol in treating IDD by focusing on ferroptosis.

#### 2.1.2 Polymeric Organic-Dominated Nanozymes

Polymeric nanozymes are synthetic or natural macromolecules with enzyme-like activity. These materials are advantageous due to their high biocompatibility, molecular imprinting capabilities, and low toxicity. They can be classified into natural (e.g., PDA, chitosan) and synthetic polymers (e.g., polyacrylamide, polyethylene glycol-functionalized systems).

PDA, inspired by mussel-adhesive proteins, displays intrinsic POD and SOD-like activities. Its catechol and amine groups can participate in redox reactions and free-radical scavenging, useful in neuroprotection and anti-inflammatory applications (Figure 4D).^98,99^

Moreover, MIPs are engineered to have specific recognition sites, functioning as artificial enzymes with substrate selectivity. These MIP nanozymes can mimic protease or esterase activity, especially when tailored with active site mimics and cofactors.^100,101^

Synthetic polymer nanozymes are also engineered by incorporating catalytic groups or redox-active centers into a polymer backbone, yielding materials with tailored enzyme-like kinetics and substrate preferences.

#### 2.1.3 Supramolecular and Peptide-Based Nanozymes

Supramolecular assemblies composed of peptides, amphiphiles, or other self-assembling organic molecules can form nanostructures that mimic the enzymes’ active sites. These nanozymes often rely on noncovalent interactions such as hydrogen bonding, π–π stacking, and hydrophobic effects to organize catalytic domains.^102^ These nanozymes are formed from self-assembling peptides or amphiphiles, often without metal ions, relying instead on the catalytic activity of amino acid residues (e.g., histidine, serine). Their function is derived from biomimetic design, hydrogen bonding, and π–π stacking—not from metal centers.

Peptide-based nanozymes utilize the chemical versatility of amino acids and can form nanofibers, nanotubes, or hydrogels with catalytic functions. Histidine- or serine-rich peptides can mimic hydrolase or POD enzymes by providing nucleophilic or redox-active residues at the catalytic core.^103,104^

Such biomimetic systems are particularly useful in medical diagnostics, biosensing, and tissue engineering, due to their structural and functional resemblance to natural enzymes and excellent compatibility with biological systems.

### 2.2 Organic Framework-Based Nanozymes

Organic framework-based nanozymes are constructed from well-defined porous structures such as COFs, MOFs with dominant organic content, or MIPs. These materials offer high surface area, chemical tunability, and customizable pore environments for mimicking enzyme active sites. Their periodic architectures facilitate efficient substrate diffusion and catalysis, making them promising candidates for biosensing, pollutant degradation, and smart agricultural technologies.

#### 2.2.1 COF-Derived Organic-Dominated Nanozymes

COFs are 2D or 3D crystalline polymers formed by the covalent linkage of organic building blocks, typically incorporating boronic acid, imine, triazine, or hydrazone linkages. Their periodic porosity, high surface area, and tunable chemistry make them excellent candidates for enzyme mimicry.

Certain COFs, particularly those containing metalloporphyrin or metallophthalocyanine units, exhibit OXD-like or POD-like activity, functioning analogously to natural enzymes such as horseradish peroxidase (HRP) (Figure 4B).^105,107^ Their porous and crystalline architecture allows efficient substrate diffusion and active site exposure, enhancing catalytic performance.^108^

Additionally, COFs can be designed as photoresponsive nanozymes, enabling light-triggered activation, a feature not available in most inorganic systems. This makes them especially useful in photobiosensing and photodynamic therapy.^82,109^

#### 2.2.2 MOF-Derived Organic-Dominated Nanozymes

MOFs are crystalline porous materials composed of organic ligands and metal ion nodes, bridging the properties of organic frameworks and metal centers. MOFs are best classified as hybrid nanozymes because they consist of both inorganic metal ions or clusters and organic ligands that form a porous crystalline structure. This dual composition gives MOFs unique catalytic properties that mimic enzyme active sites. While some classification systems might simplify nanozymes into organic or inorganic categories, MOFs do not fit neatly into either group. They are often grouped under inorganic nanozymes due to the dominant role of metal centers in catalysis, but their organic linkers also contribute significantly to their function. Therefore, referring to MOF-based nanozymes as hybrid nanozymes is the most accurate and scientifically appropriate classification, reflecting their combined organic–inorganic nature and the complexity of their catalytic mechanisms.

Porphyrin-based MOFs, in particular, are excellent models of nature’s metalloenzymes (e.g., cytochrome P450).^110^ The catalytic activity arises from π-conjugated macrocyclic ligands and the accessible active metal centers. These nanozymes can catalyze oxidation, peroxidation, and even biomimetic transformations such as hydroxylation.

In addition, carbonized MOFs (e.g., ZIF-8-derived porous carbon) retain the hierarchical structure of the parent MOF and exhibit enhanced stability and conductivity, making them suitable for biosensing and environmental catalysis (Figure 4C).^106,111^

Despite their tunable structure and catalytic potential, many MOF-based nanozymes exhibit poor aqueous stability. Exposure to moisture can compromise the integrity of coordination bonds, leading to framework collapse and potential leaching of metal ions, such as Zn^2+^, Cu^2+^, or Fe^3+^.^112^ These issues may diminish catalytic activity and introduce environmental risks through ion accumulation in soil and water. Compared to metal oxides, MOFs often exhibit lower stability under real-world agricultural conditions.^113^ Therefore, enhancing water stability through hydrophobic functionalization, postsynthetic modification, or the development of water-stable MOFs (e.g., Zr-based or phosphonate-linked frameworks)^114–116^ remains a key area of ongoing research.

### 2.3 Recent Advancements in Organic-Dominated Nanozyme Research

Integrating nanozymes into agricultural practices represents an exciting development in the pursuit of sustainable food production. These artificial catalysts offer innovative solutions to longstanding challenges in crop protection, nutrient management, and environmental monitoring within agricultural ecosystems.^35^ For instance, nanozymes with POD-like activity have been employed in the development of pesticide-free crop protection strategies, catalyzing the generation of reactive oxygen species to combat pathogenic microorganisms.

Furthermore, nanozymes are being explored for their potential to enhance nutrient uptake efficiency in crops. Cerium oxide nanozymes, for example, have shown promise in promoting plant growth and stress resistance by modulating reactive oxygen species levels in plant tissues.^117^ This approach could lead to more efficient use of nutrients and reduced reliance on conventional fertilizers, contributing to more sustainable agricultural practices. Another promising area of research is using organic-dominated nanozymes in improving plant stress tolerance. By mimicking natural antioxidant enzymes, these nanozymes can help plants cope with various environmental stresses such as drought, salinity, and extreme temperatures (Figure 5A). This application could lead to the development of more resilient crop varieties capable of thriving in challenging environmental conditions.^44,118^

**Figure 5:**
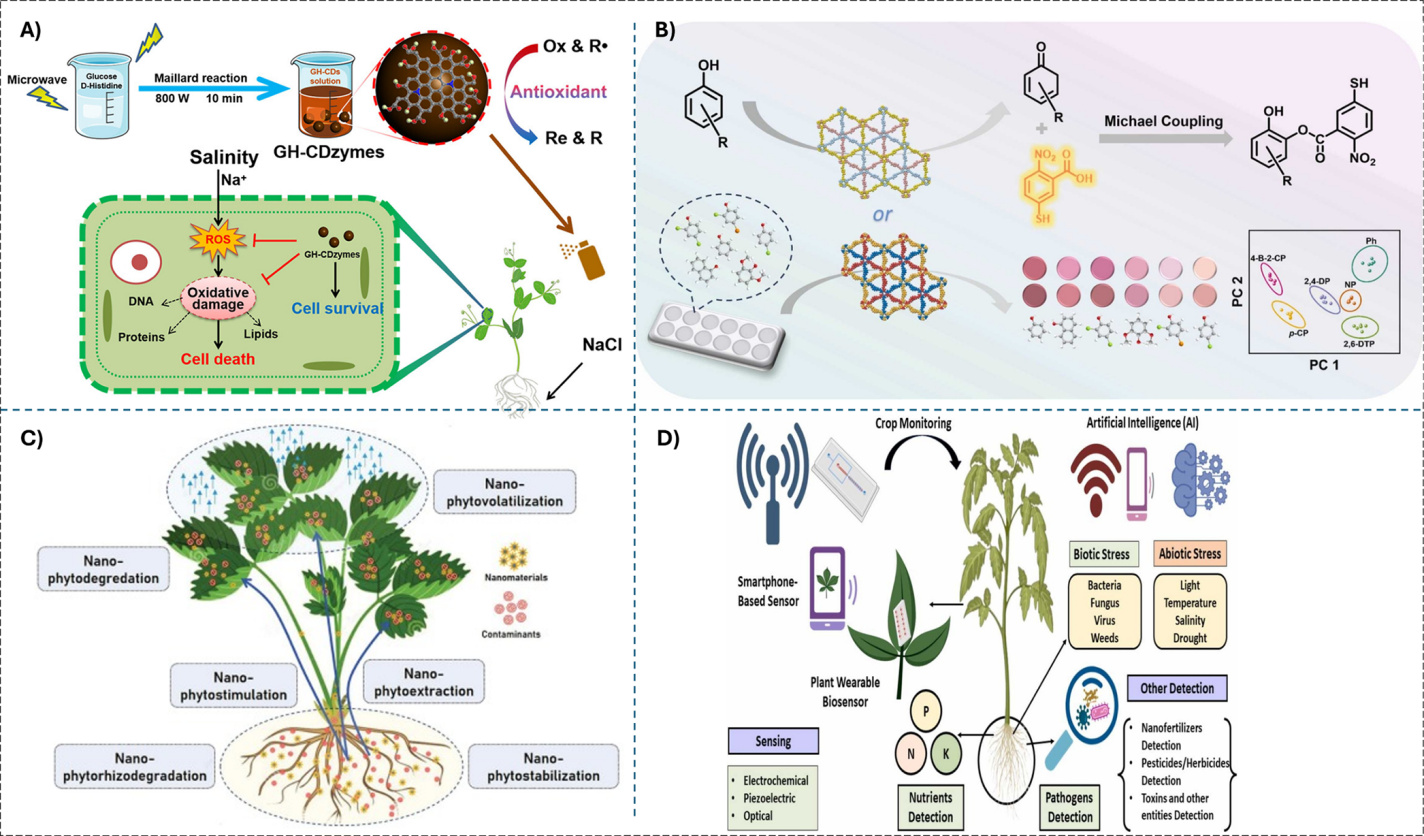
A) Schematic illustration of rational design and preparation of GH-CD nanozyme for treating salt-induced oxidative damage in plant growth. Reprinted with permission from ref. 88. Copyright (2023) American Chemical Society. B) LAC-like Cu_3_-TDH COF and Cu_3_-BDU COF with oxidative degradation ability and a dual-channel colorimetric sensor array for the identification of phenolic pollutants. Reprinted with permission from ref. 124. Copyright (2025) Elsevier. C) An illustration depicting the array of possible mechanisms of nanozyme-phytoremediation approach. Reprinted with permission from ref. 125. Copyright (2022) Elsevier. D) Applications of nanozyme-based biosensors for the detection of various parameters of plants that promote smart and sustainable agriculture. Reprinted with permission from ref. 126. Copyright (2024) Elsevier.

The field of organic-dominated nanozyme technology is also witnessing advancements in the design of multifunctional nanozymes. These advanced catalysts can perform multiple enzyme-like activities simultaneously, offering more comprehensive solutions to complex agricultural challenges. For instance, a single nanozyme could catalyze the degradation of pesticide residues while also promoting nutrient uptake in plants.^7^ Researchers are also exploring the development of stimuli-responsive organic-dominated nanozymes.^119^ These innovative materials can be designed to activate or deactivate their catalytic properties in response to specific environmental triggers such as pH, temperature, or light. This feature allows for precise control over nanozyme activity, enabling targeted applications in various agricultural contexts. The integration of organic-dominated nanozymes with other emerging technologies is another exciting frontier. For example, combining nanozymes with controlled release systems could lead to developing innovative fertilizers that release nutrients in response to specific plant needs or environmental conditions. Similarly, incorporating nanozymes into agricultural biotechnology could result in novel crop protection strategies that are both effective and environmentally friendly.

Despite the remarkable progress in organic-dominated nanozyme research and development, several challenges remain to be addressed. The optimization of catalytic specificity and efficiency, particularly in complex biological environments, continues to be an active area of investigation.^38^ Additionally, concerns regarding the potential long-term environmental and health impacts of nanozymes necessitate comprehensive toxicological studies and the development of biodegradable or easily recoverable nanozyme systems.^35^ Looking ahead, the field of nanozyme technology is poised for further expansion and refinement. Emerging trends include the development of stimuli-responsive nanozymes capable of on-demand activation, the integration of artificial intelligence for rational design and optimization of nanozyme structures, and exploring synergistic effects in multienzyme nanozyme systems.^38^ These advancements are expected to broaden the application spectrum of nanozymes and enhance their performance in existing domains.

As research in organic-dominated nanozymes progresses, a growing focus is on developing more sustainable and eco-friendly synthesis methods. Green chemistry approaches are being explored to minimize the use of harsh chemicals and reduce energy consumption during nanozyme production. This aligns with the overarching goal of creating sustainable agriculture and environmental management solutions. The potential of organic-dominated nanozymes in precision agriculture is particularly promising. By developing nanozymes that can respond to specific plant signals or environmental cues, researchers aim to create smart agricultural systems that deliver nutrients, control pests, or mitigate stress factors with unprecedented precision. This could lead to significant improvements in crop yields while minimizing the environmental impact of agricultural practices.

In the context of soil health and remediation, organic-dominated nanozymes are being investigated for their ability to enhance soil microbial activity and degrade POPs (Figure 5B). By mimicking the activities of soil enzymes, these nanozymes could play a crucial role in maintaining soil fertility and biodiversity, which are essential for sustainable agriculture.^120,121^ The application of organic-dominated nanozymes in postharvest technology is another area of growing interest. Researchers are exploring the use of these artificial enzymes to extend the shelf life of fruits and vegetables, reduce food waste, and improve food safety. For instance, nanozymes with antimicrobial properties could be incorporated into packaging materials to prevent spoilage and contamination.^67,122^

One of the most exciting developments in organic-dominated nanozyme research is the creation of multifunctional nanozymes. These advanced catalysts can perform multiple enzyme-like activities simultaneously, offering more comprehensive solutions to complex agricultural challenges. For example, a single nanozyme could potentially catalyze the degradation of pesticide residues while also promoting nutrient uptake in plants.^122^ The integration of organic-dominated nanozymes with other emerging technologies opens up new possibilities for sustainable agriculture.^123^

Combining nanozymes with controlled release systems could lead to the development of smart fertilizers that release nutrients in response to specific plant needs or environmental conditions. Similarly, incorporating nanozymes into agricultural biotechnology could result in novel crop protection strategies that are both effective and environmentally friendly.^127,128^ Researchers are also exploring the potential of organic-dominated nanozymes in phytoremediation, where plants are used to remove contaminants from soil and water (Figure 5C). By enhancing the plants’ natural ability to break down pollutants, nanozymes could significantly improve the efficiency of these remediation efforts.

Another promising area of research is the development of nanozyme-based biosensors for real-time monitoring of soil and plant health (Figure 5D). These sensors could provide farmers with valuable data on nutrient levels, pest infestations, or plant stress, allowing for more timely and targeted interventions.^129,130^ As the field of organic-dominated nanozymes continues to evolve, researchers are also addressing potential challenges and limitations. One key area of focus is improving the stability and longevity of organic-dominated nanozymes in complex environmental conditions. This includes developing strategies to protect the nanozymes from degradation and maintain their catalytic activity over extended periods.^131^

The scalability of organic-dominated nanozyme production is another important consideration for their widespread adoption in agriculture. Researchers are developing cost-effective and easily scalable synthesis methods that can meet the demands of large-scale agricultural applications.^83,123,128^ Safety and regulatory aspects of organic-dominated nanozymes in agriculture are also receiving increased attention.^128^ Comprehensive studies are being conducted to assess the potential long-term effects of these materials on ecosystems and human health. This research is crucial for establishing guidelines and regulations for the responsible use of nanozymes in agricultural settings.^35,132^ Ultimately, organic-dominated nanozymes for sustainable agriculture are rapidly advancing, offering innovative solutions to longstanding challenges in crop production, environmental protection, and food security. As research continues to progress, these artificial enzymes have the potential to revolutionize agricultural practices, leading to more efficient, sustainable, and environmentally friendly food production systems. The ongoing exploration of novel nanozyme compositions, structures, and functionalities promises to unlock even more significant potential in environmental remediation, precision agriculture, and advanced agricultural technologies. This article examines nanozymes composed of organic materials, a category that gained formal recognition in the 2020s. Additionally, it explores the possible uses and prospects of organic-dominated nanozymes in promoting sustainable agricultural practices.

## 3 Applications of Organic-Dominated Nanozymes in Sustainable Agriculture

The applications of organic-dominated nanozymes span several crucial aspects of agricultural sustainability, including the detection of herbicides and pesticides, soil health monitoring, and the enhancement of crop growth and protection. By leveraging their unique enzymatic properties, organic-dominated nanozymes minimize environmental contamination, promote soil fertility, and improve agricultural productivity while reducing reliance on synthetic chemicals.

One of the most pressing challenges in modern agriculture is the overuse of chemical herbicides and pesticides, which pose significant environmental and health risks. Organic-dominated nanozymes have shown great potential in the rapid and sensitive detection of these agrochemicals, ensuring food safety and minimizing ecological damage. Functionalized nanozymes with POD- or OXD-like activities can catalytically amplify colorimetric, fluorometric, or electrochemical signals, allowing for the real-time monitoring of pesticide residues in crops, soil, and water.^6^ For instance, nanozyme-based biosensors have been developed to detect organophosphates and carbamates with high specificity and low detection limits.^133^ Compared to conventional detection methods such as chromatography or mass spectrometry, nanozyme-based sensors offer a more affordable, portable, and user-friendly solution, enabling farmers and regulatory agencies to conduct on-site analysis with minimal technical expertise. This real-time monitoring capability plays a crucial role in reducing the excessive application of agrochemicals and mitigating their adverse effects on ecosystems.

Beyond agrochemical detection, organic-dominated nanozymes are also pivotal in soil health monitoring and improvement. Soil degradation, often caused by intensive farming practices, threatens global food security by depleting essential nutrients and disrupting microbial ecosystems. Nanozymes with OXD- or SOD-like activity can act as bioindicators of soil oxidative stress, providing valuable insights into microbial activity and nutrient cycling.^134,135^ Moreover, nanozymes can be engineered to facilitate bioremediation by catalyzing the degradation of POPs and heavy metals in contaminated soils.^136^ For example, lignin-derived organic-dominated nanozymes have demonstrated efficacy in breaking down toxic phenolic compounds and improving soil microbiome diversity.^137,138^ Additionally, the application of nanozymes can enhance nitrogen fixation and phosphorus solubilization, crucial processes for maintaining soil fertility.^139,140^ These advancements contribute to the development of precision agriculture techniques that optimize resource use while preserving environmental integrity.

Enhancing crop growth and protection is another promising application of organic-dominated nanozymes in sustainable agriculture. Nanozyme-based fertilizers and plant growth stimulants can mimic natural enzymatic pathways to enhance crop nutrient uptake and stress resistance. For instance, nanozymes with CAT-like properties help mitigate oxidative stress in plants, improving their resilience to drought, salinity, and pathogen attacks.^141^ Furthermore, organic-dominated nanozymes have been explored as antimicrobial agents to combat plant pathogens without using synthetic fungicides or bactericides.^142,143^ Their catalytic mechanisms enable the targeted generation of ROS to disrupt microbial cell membranes while minimizing harm to beneficial soil microbiota. Additionally, encapsulated nanozyme formulations can be employed for controlled and sustained nutrient release, ensuring that plants receive essential micronutrients over extended periods, thereby reducing fertilizer wastage and runoff pollution.

Overall, organic-dominated nanozymes represent a revolutionary approach to achieving sustainability in agriculture. Their multifunctional capabilities in agrochemical detection, soil health management, and crop protection align with precision and eco-friendly farming principles. By integrating nanozyme-based technologies into agricultural practices, it becomes possible to reduce chemical inputs, enhance productivity, and promote environmental stewardship. As research in this field progresses, further innovations in organic-dominated nanozyme design and application will likely pave the way for a more resilient and sustainable global food system.

Most research on organic-dominated nanozymes in agriculture has primarily focused on their application in pesticide sensing, leveraging their enzyme-mimicking properties for rapid and sensitive detection. These studies have demonstrated significant advancements in developing nanozyme-based biosensors for real-time monitoring of pesticide residues in soil, water, and crops. However, other emerging applications, such as soil health improvement, crop protection, and nutrient enhancement, are still in their early stages, with limited experimental validation and practical deployment. Given the extensive progress in pesticide detection, our focus will remain on this well-established area while acknowledging the potential of organic-dominated nanozymes in broader agricultural sustainability.

### 3.1 Organic-Dominated Nanozyme for Pesticides Sensing

Pesticides are chemical substances or mixtures intended to control, prevent, and destroy pests, animals, or human disease-causing vectors, undesirable plants, or animal species.^144^ Based on their target organisms, pesticides are categorized as insecticides (organochlorine, organophosphate, carbamate, pyrethroid, etc.), herbicides (phenoxy hormone products, triazines, amides, sulfonylureas, etc.), fungicides and bactericides (inorganic, dithiocarbamate, benzimidazole, triazoles, diazoles, etc.), rodenticides (anticoagulant, cyanide generator, hypercalcaemic, narcotics, etc.), and plant growth regulator.^145^

As the world’s population and demand for food have increased, the consumption of pesticides for agriculture nearly doubled from 1990 to 2022, rising from 1.806 million tons to 3.698 million tons, among which insecticides and herbicides account for the main proportion (Figure 6A).^146^ However, pesticide residues in the environment also pose potential risks to food quality, nontarget organisms, and humans due to their persistence and toxicity.^147,148^ Therefore, it is vitally significant to develop rapid and credible detection methods for residue monitoring pesticide residue.

**Figure 6:**
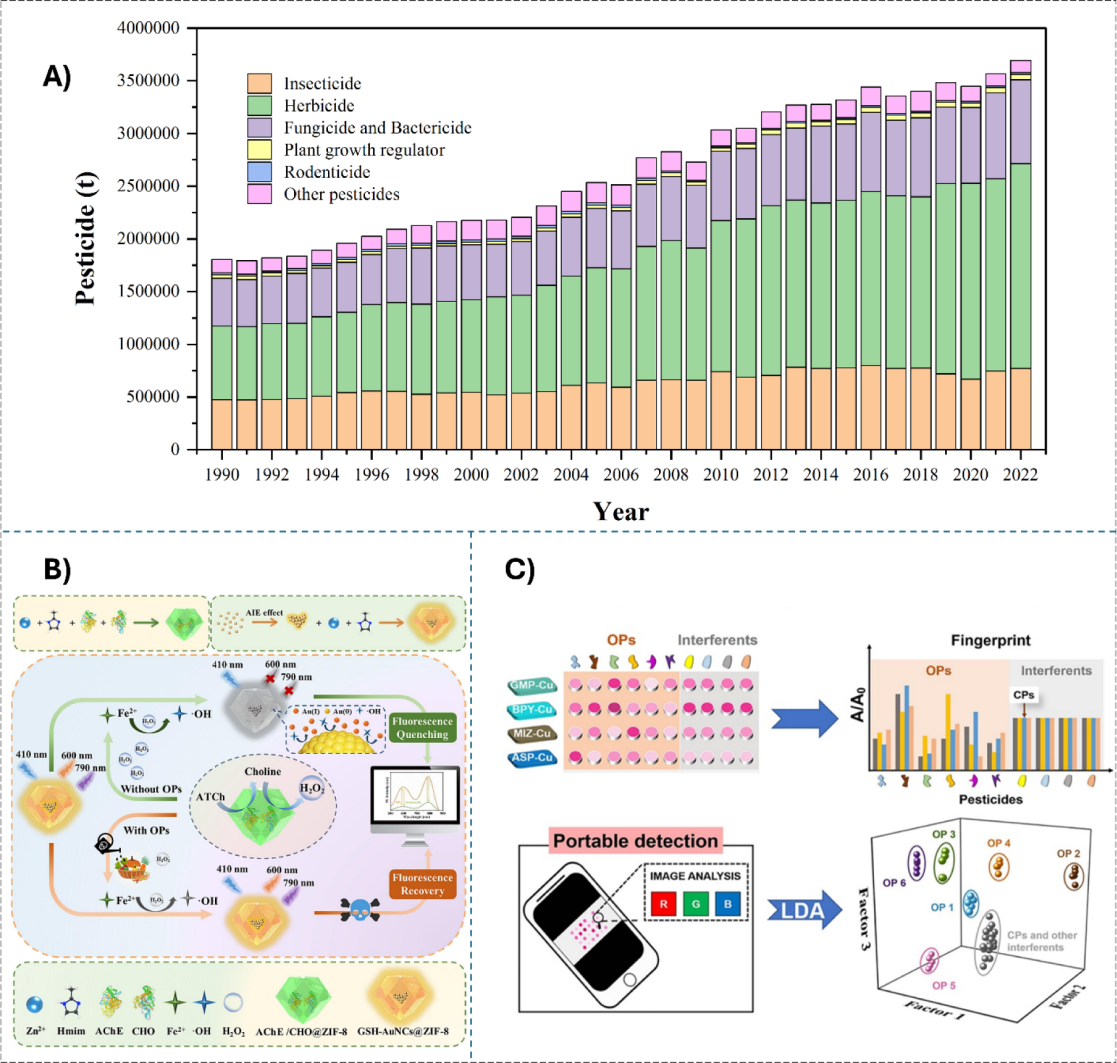
A) Worldwide pesticide consumption for agriculture use (Data retrieved from https://www.fao.org/faostat/en/#data. Retrieval date: March 18, 2025; Groups: Land, inputs and sustainability; Domains: Pesticides Use; Years from 1990–2022). B) Schematic diagram of ZIF-8-based cascade amplification nano bioreactor for the detection of methyl parathion in fruits and vegetables. Reprinted with permission from ref. 154. Copyright (2024) Elsevier. C) Nanozyme sensor array for identification of OPs from CPs, antibiotics, ions, other pesticides. Reprinted with permission from ref. 155. Copyright (2023) Elsevier.

Current approaches for pesticide residue detection encompass conventional chromatography techniques and advanced biosensors. Chromatography techniques such as gas chromatography (GC) and liquid/gas chromatography-tandem mass spectrometry (LC–MS/MS, GC–MS/MS) remain the gold-standard confirmatory methods for trace-level quantitative analysis due to their unparalleled precision and reliability.^149^ Biosensors, which utilize biological recognition elements (e.g., enzymes, antibodies, aptamers) coupled with nanomaterials (e.g., quantum dots, gold nanoclusters, and nanoparticles) to transduce interactions into measurable signals via fluorescence, colorimetric changes, or electrochemical responses, have gained significant attention for their rapid response, high sensitivity, and portability (Figure 6B).^150–153^

However, traditional biorecognition components, such as natural enzymes, suffer inherent limitations like poor stability and high cost. Nanozyme-based sensors have emerged as a revolutionary alternative, addressing these challenges through their strong enzyme-mimicking activity, remarkable stability, and cost-effectiveness.^156,157^ While inorganic nanozymes (e.g., SANs) face constraints such as complex synthesis, poor biocompatibility, and environmental concerns, organic-dominated nanozymes represent the next generation of these materials.^67^ They expand the functional scope of nanozyme technology and exhibit promising practicality for detecting organic pollutants like pesticides. To date, research has primarily focused on organophosphorus pesticides (OPs) and glyphosate. Additionally, advancements in multipesticide discrimination strategies using sensor arrays are increasingly bridging gaps in specificity and multiplexed detection capabilities.^158^ A summary of the recent advances in organic-dominated nanozymes for pesticide sensing is given in Table 2. It can be noticed that carbon-based nanozymes dominate pesticide sensing due to their tunable properties.

**Table 2:** Recent Advances in Organic-Dominated Nanozymes for Pesticide Sensing: Composition, Mechanisms, and Analytical Performance

Nanozyme Composition	Target Analyte	Sensing Method	Linear Range	LOD	ref
Zr–TCPE MOF-based composite	Parathion	Colorimetric and fluorescent	1.82–181.69 μM (colorimetric); 0.36–181.69 μM (fluorescent)	0.178 μM (colorimetric); 0.195 μM (fluorescent)	^168^
Algae-derived N-doped biochar	Diafenthiuron, bensulfuron methyl, fomesafen, lactofen, and starane	Colorimetric sensor array	1–500 μM	1 μM	^169^
Metal NPs-decorated CNTs	Carbendazim, isoproturon, glyphosate, profenofos, atrazine, diethyl cyano phosphonate, deltamethrin, and diethyl phosphoramidite	Colorimetric sensor array	1–8 μM	10.8–28.8 nM	^170^
NH_2_–CuBDC MOF-based	Chlorpyrifos	Colorimetric and fluorescent	1.8–180 ng/mL (colorimetric); 4.5–450 ng/mL (fluorescent)	1.57 ng/mL (colorimetric); 2.33 ng/mL (fluorescent)	^171^
MB/COF@MnO_2_ composite	Dichlorvos	Fluorescent and electro-chemical	Not specified	0.083 ng/mL (fluorescent); 0.026 ng/mL (electrochemical)	^172^
Luminescent aluminum MOF	Paraoxon-methyl	Fluorescent	Not specified	0.3 nM	^173^
Recyclable luminescent aluminum MOF	Dinotefuran	Fluorescent	Not specified	2.3 nM	^174^
Urea and polyvinyl alcohol with Fe–N coordination	Glyphosate	Colorimetric	Not specified	0.001 ng/mL	^69^
Heteroatom-doped graphene	Lactofen, fluoroxypyr-meptyl, bensulfuron-methyl, fomesafen, and diafenthiuron	Colorimetric sensor array	5–500 μM	Not specified	^175^
Supramolecular MOF with tetra-pyridyl calix[4]arene	Glyphosate	Fluorescent	2.5–45 μM	2.25 μM	^176^

### 3.2 Sensing Strategies for Pesticides

Nanozymes have shown remarkable advantages in the application of pesticide detection, the core of which is the combination of biomimetic catalytic activity and material designability. Most pesticide sensors are based on the regulation of POD- and OXD-like activity or reaction.^159^ For example, TMB, a typical chromogenic substrate, can be converted to its oxidized state oxTMB with the characteristic absorption peak at 652 nm by OXD-like nanozyme with ambient O_2_ or POD-like nanozyme with H_2_O_2_ as an additional cosubstrate. Various pesticide sensors have been constructed by regulating the nanozyme activity, the generation of chromogenic substrates (oxTMB), and the concentration of cosubstrates. The detection strategies are mainly attributed to the following three directions.

#### 3.2.1 Enzyme–Nanozyme Cascade Reaction Strategy

The enzyme–nanozyme cascade reaction strategy leverages the catalytic interplay between natural enzymes and nanozymes to achieve highly sensitive and selective detection of OP compounds.^160^ These hybrid catalytic systems exploit the substrate-specificity of enzymes combined with the robust catalytic activity of nanozymes, enabling signal amplification through colorimetric or electrochemical responses. Depending on the reaction mechanism, these strategies can be categorized into different cascade reaction pathways, each offering unique advantages for OP detection.

##### 3.2.1.1 ATCh-AChE-Nanozyme Cascade Reaction Strategies

The SH-containing thiocholine (TCh), which is hydrolyzed from acetylthiocholine (ATCh) by acetylcholinesterase (AChE), can block the nanozyme active sites, reduce the oxidized chromogenic substrate, or reduce the nanozyme itself. Nevertheless, further investigation is needed to understand the underlying mechanism. In the absence of an AChE inhibitor such as OPs, the production of TCh is suppressed, while the production of oxTMB is increased, thus triggering an enhanced absorbance at 652 nm.^161,162^ By monitoring the absorbance or solution color change, a “turn on” colorimetric method for the detection of OPs was established.

##### 3.2.1.2 ACh-AChE-ChOx-Nanozyme Cascade Reaction Strategy

Another strategy of colorimetric sensors for OP detection is based on the ACh-AChE-ChOx-nanozyme cascade reaction. First, acetylcholine (ACh) is catalyzed to produce choline by AChE, and then choline is catalyzed to produce betaine and H_2_O_2_ by ChOx. Finally, with H_2_O_2_ as an additional cosubstrate, TMB is oxidized to blue oxTMB by nanozyme with POD-like activity. In the absence of an AChE inhibitor such as OPs, the production of H_2_O_2_ is inhibited, and the production of oxTMB is also reduced.^163^ By monitoring the absorbance or solution color change, a “turn off” colorimetric method for the detection of OPs was established.

#### 3.2.2 Metal Ion Mediated Nanozyme Activity Regulation

Metal ions may serve as catalytic active centers in organic-dominated nanozyme or stabilize nanozyme structures through coordination bonds. Due to the specific coordination and electrostatic binding interactions between metal ions with TCh^163,164^ or pesticide itself,^165^ the catalytic activity of nanozyme is modulated, generating measurable signals such as colorimetric and fluorescent signals. Metal ion-mediated regulation offers a versatile toolkit to tailor nanozyme activity, balancing sensitivity, selectivity, and practicality for pesticide detection.

#### 3.2.3 Sensor Array Strategies for Multipesticide Discrimination

Most existing sensors for pesticide detection are limited to single pesticide or specific categories, multiple pesticide analyses remain a significant challenge. A promising solution lies in sensor arrays, which leverage cross-responsive sensing elements and data dimensionality reduction techniques (e.g., principal component analysis (PCA), linear discriminant analysis (LDA), hierarchical clustering analysis (HCA), machine learning (ML)) to enable simultaneous identification and discrimination of diverse analytes.^166,167^ Recent advances highlight nanozyme materials with enzyme-like catalytic properties as ideal candidates for constructing these arrays due to their tunable activities, stability, and cost-effectiveness. organic-dominated nanozymes (e.g., heteroatom-doped graphene, algae-derived biochar, Asp-Cu complexes) offer additional advantages such as biocompatibility, streamlined synthesis, and environmental sustainability (Figure 6C). This approach addresses the limitations of traditional single-analyte methods and paves the way for high-throughput, multiplexed detection systems. The design of sensing elements is the key factor for the construction of sensor assay. The detection of multipesticides mainly relies on the following three strategies: (1) multienzyme systems as sensing elements,^155,175^ (2) single nanozyme with multienzyme activities (e.g., POD-, LAC-, and SOD-like functions) as sensing elements,^177^ and (3) multisubstrates (such as TMB, o-phenylenediamine (OPD), and 2,2’-azino-bis­(3-ethylbenzothiazoline-6-sulfonic acid)­(ABTS)) catalyzed by single nanozyme as the sensing element.^178^

### 3.3 Sensing of Conventional Organophosphorus Pesticides (OPs)

OPs, a class of insecticides extensively employed in agriculture for their high efficacy and broad-spectrum activity, play a critical role in pest management.^179^ Structurally, OPs feature a central phosphorus atom double bonded to an oxygen or sulfur atom, with two alkoxy groups (R1 and R2) and a leaving group (X) completing the tetrahedral arrangement. Their insecticidal action stems from the irreversible inhibition of AChE, an enzyme vital for neurotransmission in insects and nontarget organisms, leading to lethal neurotoxic effects. By monitoring the nanozyme activity or enzyme-nanozyme-cascade reaction, various biosensors, such as optical colorimetric, fluorometric, and electrochemical platforms, have been developed for the detection of OPs. Notably, organic material-based nanozymes, such as heteroatom-doped and metal-doped carbon variants, have shown promise in enhancing detection accuracy while addressing limitations of traditional inorganic nanozymes, such as biocompatibility and synthesis complexity.

#### 3.3.1 Carbon and COF Nanozyme

Based on enzyme–nanozyme cascade reaction mediated oxTMB consumption strategy, Zhang et al.^161^ developed a metal-free N-doped carbon nanozyme (NC900) via the direct high-temperature pyrolysis of a nitrogen-rich conjugated microporous polymer for the screen of OPs such as Dursban (chlorpyrifos). The NC900 has robust OXD-like activity (*K*_m_ = 0.26 mM, *V*_max_ = 1.52 × 10^–7^ M/s), anti-interference features (colorless), and broad operational stability (wide temperature range and long-term stability). TMB was converted to its oxidized state (oxTMB) in the presence of NC900 and was reduced by the AChE-ATCh reaction generated TCh. Based on AChE inhibition of OPs, a colorimetric assay for screen of OPs was established. Liang et al.^162^ constructed a COF nanozyme (TpBTD), which can exhibit outstanding OXD-like activity under flashlight illumination. Using a smartphone as a light source and colorimetric RGB readout device, a point-of-care testing (POCT) platform for the detection of OPs such as trichlorfon was developed via inhibition of the AChE-ATCh reaction and increase of TMB oxide reaction (Figure 7A).

**Figure 7:**
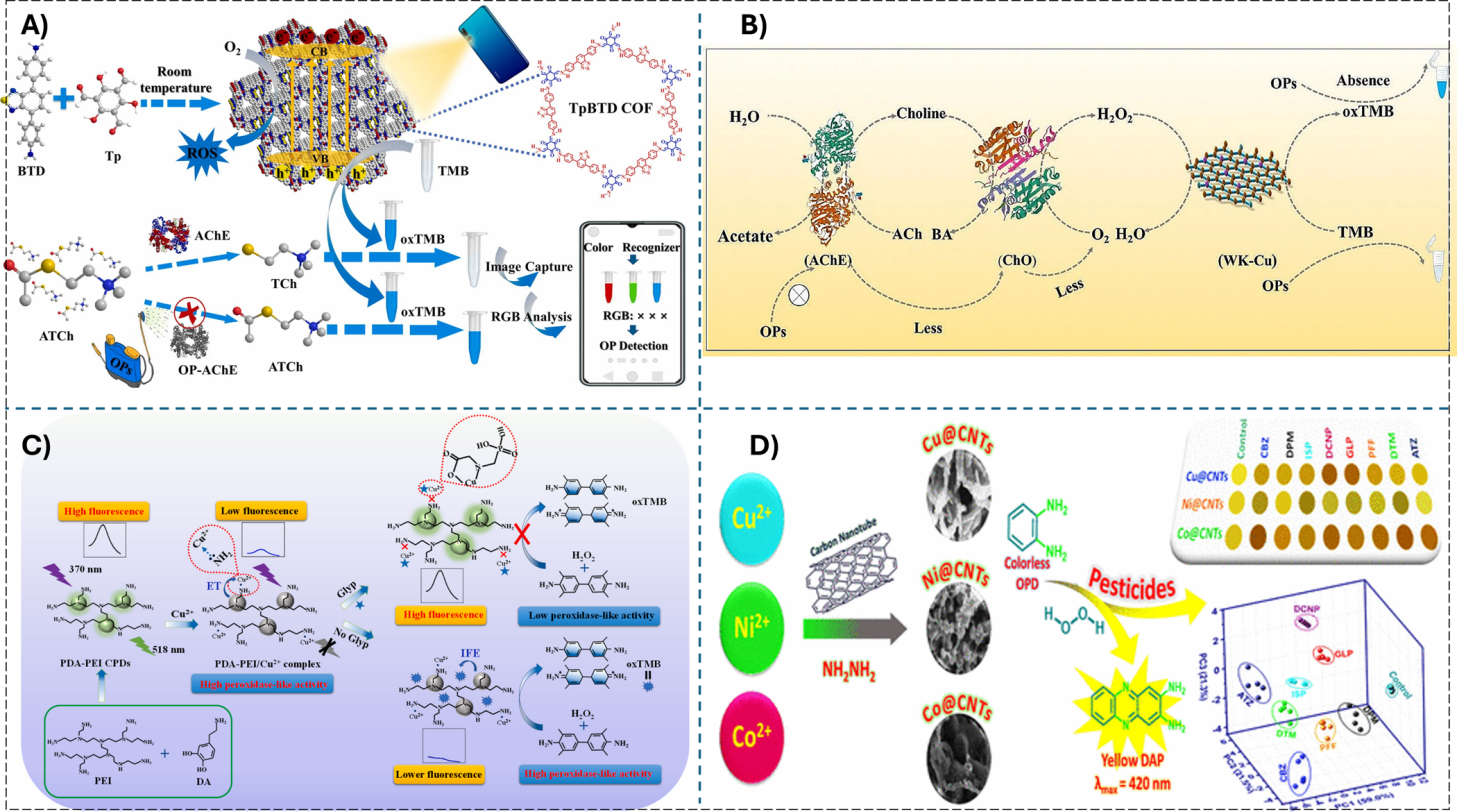
A) Smartphone flashlight-triggered the oxidase-mimicking activity of TpBTD and the visual POCT platform for OPs assay. Reprinted with permission from ref. 162. Copyright (2023) Elsevier. B) Colorimetric method for OPs detection using wool keratin- and Cu-based multienzyme cascade reaction. Reprinted with permission from ref. 163. Copyright (2024) Springer Nature. C) Cu (II)- containing PDA–PEI CPDs for dual-mode colorimetric-fluorescent detection of glyphosate in the environment. Reprinted with permission from ref. 182. Copyright (2023) Elsevier. D) the Development of the MDC-based nanozyme sensor array for discrimination of pesticides. Reprinted with permission from ref. 170. Copyright (2024) American Chemical Society.

#### 3.3.2 Metal-Coordinated Organic-Dominated Nanozyme

Beyond carbon-based nanozymes, small metal ions (such as Cu^2+^, Fe^2+^, and Co^2+^) coordinated organic-dominated nanozymes are utilized to fabricate pesticide sensors. These metal ions provide active sites for the generation of nanozymes and sensing recognition sites for pesticides sensing.

For instance, Cao et al.^163^ synthesized a wool keratin–copper (WK–Cu) nanozyme via self-assembly, demonstrating POD-like activity in multienzyme cascade reactions. OPs like phoxim inhibit AChE, reducing H_2_O_2_ generation and oxTMB production (Figure 7B). The method achieved a wide detection range, low LOD, and reliable performance in complex food matrices (apple, cabbage, cauliflower). Additionally, Bhatt et al.^164^ engineered a Cu^2+^-coordinated supramolecular nanozyme (SupraZyme) with intrinsic OXD-like activity, catalyzing the formation of a pink quinone–imine complex (absorbance at 510 nm) from 2,4-DP and 4-AP. Thiol-containing biomolecules (TCh) inhibit >90% of its activity via Cu–S bond formation, enabling selective detection of OPs.

While most nanozyme-based sensors rely on catalytic inhibition, Yang et al.^165^ reported a unique enhancement mechanism. Dichlorvos strengthens the catalytic activity of CDs–Fe nanozymes through electrostatic attraction and π–π stacking, improving substrate affinity and electron transfer efficiency. This contrasts with traditional inhibition-based strategies, expanding the toolkit for pesticide sensing.

### 3.4 Sensing of Glyphosate

Glyphosate, an OP compound, is a broad-spectrum herbicide. Unlike OPs, it inhibits 5-enolpyruvylshikimate-3-phosphate synthase (EPSPS), a key enzyme in the shikimate pathway responsible for synthesizing aromatic amino acids (e.g., tyrosine, phenylalanine) in plants, bacteria, and fungi.^180^ The widespread use of glyphosate since its commercialization in 1974 has brought risks of crop resistance and toxicity to fish and mammal health.^181^ Therefore, rapid detection of glyphosate is critical for mitigating risks of contamination events, ultimately supporting sustainability in agriculture and public health initiatives. While glyphosate is an OP compound, it is addressed separately due to its distinct sensing challenges and applications as an herbicide (rather than an insecticide, like most OPs).

The sensing mechanism for glyphosate is mainly due to the specifically binding or destroying the active site of nanozyme (such as metal centers or functional groups), mediating its catalytic ability and resulting in signal change. By introducing Cu^2+^ into polydopamine-polyethylenimine copolymer dots (PDA–PEI CPDs), Li et al.^182^ fabricated a PDA–PEI/Cu^2+^ complex possessing both POD-mimetic activity and stimulus-responsive fluorescence. Due to the coordination between glyphosate and Cu^2+^-, the Cu^2+^-induced fluorescence quenching can be significantly recovered meanwhile the POD-mimicking activity be strongly hindered. By recording the “turn on” fluorescence intensity of PDA–PEI/Cu^2+^ and the “turn off” colorimetric intensity of oxTMB, a dual-mode platform for sensing glyphosate was established (Figure 7C).

Another potential sensing mechanism may be attributed to the radical scavenging activity of glyphosate. Lee and Kamruzzaman^69^ synthesized an organic nanozyme with a POD-like nanozyme by chelating iron ions with urea and poly­(vinyl alcohol) in one pot. The nanozyme exhibits stable activity in a wide range of pH (2–8.5) for the catalysis of ABTS. The presence of glyphosate can decrease the absorbance of oxABTS, which may attribute its radical scavenging activity to the oxidation of ABTS.

### 3.5 Sensor Arrays for Discrimination of Multipesticides

Organic-dominated nanozymes, such as heteroatom-doped graphene and algae-derived biochar address the limitations of traditional single-analyte methods and pave the way for high-throughput, multiplexed detection systems.

#### 3.5.1 Heteroatom-Doped and Metal Ion-Decorated Carbon Nanozyme

Carbon materials, including GO, CNTs, and carbon nanospheres (CNSs), have demonstrated POD-like catalytic activities. Doping with heteroatoms such as N and S modifies their electronic structure and electric field distribution, optimizing adsorption energy barriers and enhancing catalytic performance. These tunable properties make heteroatom-doped carbon nanozymes ideal for constructing sensor arrays to detect and discriminate pesticides. For example, Zhu et al.^175^ developed a sensor array using nitrogen-doped graphene (NG), nitrogen/sulfur codoped graphene (NSG), and GO. Aromatic pesticides inhibit the POD-like activity of graphene by blocking its active sites via π–π stacking and hydrogen bonding. Colorimetric responses were analyzed by LDA, achieving clear separation of five aromatic pesticides (lactofen, fluoroxypyr-meptyl, bensulfuron-methyl, fomesafen, and diafenthiuron) into distinct clusters in 2D score plots. Yue et al.^169^ fabricated nanozyme arrays from N-doped biochar derived from *Spirulina*, *Chlorella*, and *Enteromorpha*. Most pesticides inhibited POD-like activity, enabling discrimination via HCA. LDA further confirmed the sensor’s ability to distinguish single pesticides from mixtures in soil, water, and food samples with high accuracy in real-world applications.

Beyond heteroatom doping, metal cation decoration is another strategy for mediating carbon-based nanozymes. For instance, Cu-, Ni-, and Co-decorated CNTs with POD-like activity were synthesized to construct a sensor array.^170^ These metal-decorated carbon (MDC) nanozymes interact with pesticides via cation−π, π–π stacking, and hydrogen bonding, modulating their catalytic activity and inducing distinct color changes in OPD. PCA and HCA demonstrated a clear separation of eight pesticides into nonoverlapping clusters, highlighting the discriminatory power of the array (Figure 7D).

#### 3.5.2 Cu-Based Organic-Dominated Nanozymes

Organic-dominated nanozymes, which integrate metallic ions such as Fe and Cu with organic materials (polymers, cellulose, urea, DNA, etc.), are regarded as next generation nanozymes due to their simple preparation, low price, good biocompatibility, and environmental sustainability. Cu-based nanozyme offers the benefits of high biocompatibility and a range of sensor arrays for discrimination based on their distinct multienzyme mimicking activities.

Based on the metal–ligand interaction between Cu with different ligands, four nanozymes LAC-like activity including guanosine 5′-monophosphate-copper (GMP-Cu), 4,4′-bipyridine-copper (BPY-Cu), 2-methylimidazole-copper (MIZ-Cu), and l-aspartic acid-copper (ASP-Cu) were prepared and used for construction of a four-channel sensor assay. Due to the special binding of the phosphate ester to the metal center Cu­(II), a sensor array for the specific recognition of OPs was established. A smartphone-assisted portable method validates its practicality in fruits and vegetables, emphasizing field applicability.

In contrast to muti-nanozyme arrays, multifunction single nanozymes address the cost and complexity challenges. For example, based on the LAC-, POD-, and SOD-like activities of Asp-Cu, a three-channel sensor array was constructed to discriminate eight pesticides (glyphosate, phosmet, isocarbophos, carbaryl, pentachloronitrobenzene, metsulfuronmethyl, etoxazole, and 2-methyl-4-chlorophenoxyacetic acid) across multiple categories.^177^ Due to the unique interactions between the functional groups in different pesticides with Asp-Cu, the accessibility of the substrate to the active site is affected, resulting in differential changes in the enzymatic activities of Asp-Cu. By handling LDA and HCA, the reliability of distinguishing diverse pesticides of the sensor array was evaluated. The results showed that the array achieves 100% accuracy in identifying unknown samples through a concentration-independent model. The system’s robustness in real samples and resistance to interference highlight its potential for food safety monitoring. Similarly, based on the unique interactions between pesticides with Cu^2+^ coordinated 2-aminoterephthalic acid (Cu-BDC-NH_2_) integrates LAC-like activity, POD-like activity, and intrinsic fluorescence, a multisignal sensor assay was constructed for cross-reactive distinguish of six pesticides classes.^183^

Conventional pesticide sensor assays are based on enzyme/nanozyme inhibition and mathematical analysis, often vulnerable to concentration-dependent signal variability and complicating precise differentiation. ML decodes hidden patterns by bridging the gap between raw signals and interpretable results. By combining a Mel-Cu nanozyme (with LAC/POD activities) and cholinesterases (AChE and BChE), a four-channel array was constructed.^184^ Via ML algorithms, 12 pesticides were enabled for concentration-independent identification and quantification. This integration of ML addresses the limitations of static enzyme inhibition methods, offering a leap toward intelligent, high-throughput pesticide analysis with applications in complex environments.

Most sensor arrays are based on the inhibition activity of nanozyme induced by pesticides. It is worth noting that sulfonylurea pesticides (SUs) showed enhancement LAC-mimic and POD-mimic activities on ASP-Cu, BPY-Cu, and GMP-Cu, enabling a six-channel array for selective SUs recognition.^185^

These Cu-based sensor arrays collectively advance pesticide detection using nanozymes with different ligands or single nanozymes endowed with multienzyme or multisignal properties. By refining nanozyme versatility, prioritizing smartphone field readiness, integrating ML intelligent analytics, and prioritizing field readiness, organic nanozyme-based sensor arrays demonstrate the progression toward precision, adaptability, and real-sample applicability.

## 4 Challenges and Future Directions

Organic-dominated nanozymes hold great promise across various fields, including catalysis, sensing, medicine, and agriculture. Despite their potential, several challenges must be addressed to facilitate their widespread adoption and practical application. One of the foremost challenges with organic-dominated nanozymes is their stability and longevity. Unlike inorganic nanozymes, which often exhibit higher stability under harsh conditions, organic-dominated nanozymes can degrade or lose activity over time.^186^ Agricultural environments have many variations and can involve elements such as salinity, changes in pH, UV radiation, humidity, temperature differences, and many types of soil. Organic-dominated nanozymes need to be effective across these diverse conditions. The fact that these catalysts need to remain active and stable in various conditions is a major concern. Research is required to adapt nanozymes to specific agricultural conditions and to improve their resilience.^187^ In addition, the synthesis of organic-dominated nanozymes often involves complex and labor-intensive procedures that could be more easily scalable. Although small-scale laboratory synthesis can yield high-quality nanozymes, translating these processes to larger scales while maintaining consistency and performance is a significant hurdle. Developing cost-effective and scalable synthesis methods is essential for making organic-dominated nanozymes commercially viable.

To enhance the scalability of organic-dominated nanozymes for sustainable agriculture, green synthesis techniques and interdisciplinary collaborations are critical. Green synthesis methods, such as using biomass-derived precursors like lignin or cellulose, aqueous-based or solvent-free synthesis, and one-pot or microwave-assisted processes, reduce costs, energy use, and environmental impact while enabling large-scale production. Continuous flow reactors and biocatalytic synthesis further streamline manufacturing, ensuring consistent quality and high throughput. Interdisciplinary collaborations can optimize these efforts: agronomists and materials scientists can design nanozymes tailored for soil remediation or nutrient delivery; chemical engineers and nanotechnologists can develop scalable production systems; environmental scientists and toxicologists can assess long-term ecological safety; data scientists and agricultural engineers can integrate nanozyme sensors with precision agriculture; and biotechnologists and soil microbiologists can enhance nanozyme compatibility with soil microbiomes. Practical steps include pilot testing, cost analysis, regulatory engagement, and industry partnerships to transition to commercial production. Challenges like precursor variability or high initial investments can be mitigated through standardized preprocessing and funding support. By combining these strategies, organic-dominated nanozymes can be scaled to deliver cost-effective, eco-friendly solutions for pesticide sensing, soil health, and crop protection, advancing sustainable agriculture.

Consistency in catalytic performance is vital for the adoption of organic-dominated nanozymes. The synthesis of catalysts may involve differences in the synthesis methods, reagents, and/or environmental conditions that result in variability in the catalytic efficiency and selectivity. Therefore, achieving reliable and reproducible performance is necessary to ensure that organic-dominated nanozymes can be used effectively in practical applications, where consistency is key to meeting performance standards. For agricultural applications, the safety of organic-dominated nanozymes for both the environment and human health is a significant concern.^188^ Ensuring that these nanozymes do not have unintended adverse effects on soil ecosystems, water sources, or nontarget organisms is crucial. Comprehensive studies are needed to assess their environmental impact and ensure they do not accumulate in the food chain or disrupt ecological balances.

Organic-dominated nanozymes must be compatible with existing agricultural practices and technologies. This includes their integration into current pest management systems, soil treatments, and crop management practices.^189^ Developing methods for seamless integration and ensuring that nanozymes do not interfere with other agricultural inputs or practices is necessary for their successful adoption. Moreover, scaling up the production of organic-dominated nanozymes from the laboratory to field applications poses challenges. Ensuring that nanozymes can be produced in large quantities while maintaining their quality and performance is crucial. This involves addressing issues related to synthesis methods’ reproducibility and large-scale production’s economic feasibility.

Furthermore, a deeper understanding of the mechanisms behind the catalytic activity of organic-dominated nanozymes is not only necessary but also intellectually stimulating.^190^ Insights into how these nanozymes function at the molecular level can guide the design of more effective and efficient materials. This knowledge can also help troubleshoot performance issues and optimize their use in various applications.

The field of organic-dominated nanozymes in agriculture is growing rapidly, with numerous opportunities for future research and development. Future research should focus on developing organic-dominated nanozymes that are specifically designed to function optimally in various agricultural environments. This includes engineering nanozymes to withstand extreme conditions such as high salinity, drought, and varying pH levels. Also, there is a great need to create nanozymes that can be delivered precisely to specific areas in the soil or plants where they are most needed.

Additionally, improving the stability and longevity of organic-dominated nanozymes under field conditions is crucial. Research could explore developing coatings or encapsulation techniques to protect nanozymes from environmental degradation. Likewise, investigating different formulations or carriers that enhance the persistence and effectiveness of nanozymes in soil and on plants would be of great interest.

Reducing the production costs of organic-dominated nanozymes is essential for widespread adoption. Future research could focus on optimizing synthesis methods to increase yield and reduce costs. This can be aided by identifying cheaper or more abundant materials that can be used to produce nanozymes without compromising performance. Furthermore, ensuring that organic-dominated nanozymes are safe for both the environment and human health is critical. Research in this area could include conducting comprehensive studies on the toxicity of nanozymes to nontarget organisms, including plants, animals, and humans. They can also work on investigating the degradation pathways of nanozymes in the environment to understand their long-term impact and ensure they do not accumulate in harmful concentrations.

Integrating organic-dominated nanozymes with existing precision agriculture technologies can enhance their effectiveness. Future research could explore the development of NZ delivery systems that can be controlled or activated using precision agriculture technologies, such as sensors and automated systems. Furthermore, using data analytics and ML to optimize the application of nanozymes based on real-time environmental and crop data would save time and offer more perspectives. Besides, developing clear regulatory frameworks and standards for using organic-dominated nanozymes in agriculture is crucial. Research could focus on collaborating with regulatory bodies to establish guidelines and standards for the safety and efficacy of organic-dominated nanozymes. Nonetheless, conducting field trials and pilot studies to generate data can support regulatory approval and demonstrate practical benefits.

Regulatory approval for agricultural nanozymes faces challenges due to unclear guidelines on nanomaterial safety, requiring comprehensive toxicological studies to assess biodegradability, bioaccumulation, and nontarget effects on soil microbiota and crops (refs. [^35^], [^110^]). Long-term ecological impact studies are needed to ensure compliance with environmental standards, particularly for soil and water applications. Safety assessments must evaluate human health risks from food chain exposure, necessitating collaboration with toxicologists and environmental scientists to develop standardized testing protocols. Engaging regulatory bodies early, aligning with green chemistry principles, and conducting field trials to validate safety can facilitate approval. These strategies, supported by pilot testing and industry partnerships, enable scalable, safe, and eco-friendly nanozyme solutions for pesticide sensing, soil health, and crop protection.

Fostering interdisciplinary collaboration can accelerate advancements in organic-dominated nanozymes research. Future directions might involve the formation of teams that include chemists, agronomists, environmental scientists, and engineers to address the multifaceted challenges of integrating nanozymes into agriculture. Additionally, collaboration with industry partners is needed to translate the research findings into commercially viable products and applications.

In summary, the future of organic-dominated nanozymes in agriculture is promising, with numerous opportunities for innovation and improvement, offering advances in soil health, pest management, and crop productivity. By addressing these key areas and pursuing focused research, it is possible to overcome current challenges and unlock the full potential of organic-dominated nanozymes to enhance agricultural productivity, sustainability, and environmental health. Ongoing research, development, and collaboration among scientists, regulators, and agricultural stakeholders are crucial to overcoming these obstacles and harnessing the full potential of organic-dominated nanozymes in agriculture.

The emergence of organic-dominated nanozymes as an innovative and sustainable solution for agriculture marks a paradigm shift in how we approach food production, soil health, and environmental conservation. With the increasing demand for higher agricultural yields to feed a growing global population, traditional farming methods, heavily reliant on chemical fertilizers, pesticides, and intensive land use, pose significant risks to both human health and ecosystems. In contrast, organic-dominated nanozymes offer a powerful, nature-inspired alternative that aligns with the principles of green chemistry, precision agriculture, and sustainable development.

Organic-dominated nanozymes exhibit enzyme-like catalytic activity, providing functionalities that are crucial for modern agriculture. Unlike conventional enzymes, which are often expensive, unstable under extreme environmental conditions, and limited in scalability, nanozymes offer remarkable stability, cost-effectiveness, and tunable properties. These features make them ideal candidates for agricultural applications, where efficiency, durability, and environmental compatibility are essential.

One of the most promising applications of organic-dominated nanozymes is their role in enhancing soil fertility. Traditional fertilizers, while effective in supplying essential nutrients, often lead to nutrient runoff, water pollution, and soil degradation. Nanozymes, particularly those designed to mimic enzymes such as urease, phosphatase, and oxidoreductases, can regulate nutrient cycling with higher efficiency, ensuring that plants receive essential elements in a controlled and sustainable manner. This controlled release minimizes environmental contamination and optimizes resource use, making agricultural practices more sustainable.

Moreover, nanozymes demonstrate significant potential in pest and disease management. The excessive use of synthetic pesticides has led to severe ecological imbalances, including the emergence of resistant pest species, loss of biodiversity, and contamination of food chains. organic-dominated nanozymes with antimicrobial, antifungal, and insecticidal properties offer a greener alternative to synthetic pesticides. Their ability to degrade toxins and neutralize pathogens ensures that crops remain protected while minimizing harmful residues that affect both human health and the environment.

Beyond their direct impact on crop health and productivity, organic-dominated nanozymes contribute significantly to environmental sustainability. Agricultural runoff containing excess chemical fertilizers and pesticides is a leading cause of water pollution, affecting aquatic ecosystems and human water supplies. By replacing or complementing traditional agrochemicals with nanozyme-based solutions, the risk of water contamination is significantly reduced. Additionally, nanozymes aid in the decomposition of organic pollutants, facilitating bioremediation efforts in contaminated soils and water bodies.

Economically, the integration of nanozymes into agricultural systems presents both short- and long-term advantages. While the initial development and implementation of nanozyme-based technologies may require investment in research and infrastructure, the long-term benefits, such as reduced dependency on synthetic chemicals, improved crop yields, and enhanced soil health, outweigh the costs. Furthermore, nanozyme technology aligns with global trends toward sustainable and organic farming, opening opportunities for farmers to access premium markets and meeting increasing consumer demand for eco-friendly and chemical-free produce.

Despite the promising advantages of organic-dominated nanozymes, their large-scale implementation in agriculture is still in its early stages. Several challenges must be addressed to ensure their successful integration into farming practices. One of the primary concerns is understanding the long-term ecological impact of nanozyme applications. While these nanomaterials offer many benefits, their interactions with soil microbiota, plant physiology, and the broader environment require extensive study to mitigate any unintended consequences.

Another critical challenge is scalability and cost-effectiveness. Although nanozymes are more stable and efficient than natural enzymes, their large-scale synthesis and practical deployment must be optimized to compete with existing agrochemical solutions. Continued advancements in green synthesis methods, using bioderived materials and eco-friendly production processes, will be key to making nanozyme technologies both economically viable and widely accessible to farmers.

Moreover, regulatory frameworks for nanozyme applications in agriculture are still evolving. As these materials gain traction, it is essential for policymakers to establish guidelines that ensure their safe and responsible use. Collaborative efforts between scientists, agricultural stakeholders, and regulatory agencies will be crucial in developing standards that balance innovation with environmental and human safety.

The integration of organic-dominated nanozymes into agricultural practices represents a transformative step toward achieving sustainability goals. The shift away from chemical-intensive farming toward nanozyme-assisted agriculture aligns with the global commitment to reducing carbon footprints, minimizing pollution, and enhancing food security. In addition to their immediate applications, nanozymes open new avenues for smart agriculture technologies, such as responsive fertilizers, real-time soil monitoring, and targeted pest control strategies.

As research continues to explore the full potential of organic-dominated nanozymes, interdisciplinary collaboration will be essential. Chemists, biologists, agricultural scientists, and engineers must work together to refine nanozyme formulations, improve their biocompatibility, and develop scalable solutions that benefit both small-scale farmers and large agricultural enterprises. Additionally, increased investment in public awareness and education will play a critical role in promoting the adoption of nanozyme technologies. Farmers and agricultural practitioners must be equipped with the knowledge and tools necessary to integrate these innovations into their traditional farming methods effectively.

In conclusion, organic-dominated nanozymes offer an exciting and viable solution to some of the most pressing challenges in modern agriculture. By enhancing soil health, improving nutrient efficiency, mitigating pest and disease threats, and reducing environmental pollution, these nanomaterials hold immense potential for shaping the future of sustainable farming. However, realizing this potential requires continued research, policy development, and collaboration between academia, industry, and agricultural stakeholders. With a proactive approach to innovation and sustainability, organic-dominated nanozymes can lead the agricultural sector into a new era of efficiency, resilience, and environmental responsibility.

## Abbreviations

ABTS: 2,2’-Azino-bis­(3-ethylbenzothiazoline-6-sulfonic acid)
ACh: Acetylcholine
AChE: Acetylcholinesterase
ATCh: Acetylthiocholine
BSA: Bovine Serum Albumin
CAT: Catalase
CDs: Carbon Dots
ChOx: Choline Oxidase
CNTs: Carbon Nanotubes
COF: Covalent Organic Framework
DHB: Dihydroxybenzaldehyde
EPSPS: 5-Enolpyruvylshikimate-3-phosphate synthase
GC: Gas Chromatography
GO: Graphene Oxide
**g-C**_**3**_ **N**_**4**_: Graphitic Carbon nitride
HCA: Hierarchical Clustering Analysis
LAC: Laccase
LDA: Linear Discriminant Analysis
LD50: Median Lethal Dose
LC–MS/MS: Liquid Chromatography–Tandem Mass Spectrometry
ML: Machine Learning
MOFs: Metal–Organic Frameworks
MIPs: Molecularly Imprinted polymers
NG: Nitrogen-Doped Graphene
NSG: Nitrogen/Sulfur Codoped Graphene
OPD: *o*-Phenylenediamine
OPs: Organophosphorus Pesticides
OXD: Oxidase
PCA: Principal Component Analysis
PDA: Polydopamine
PEI: Polyethylenimine
POD: Peroxidase
POCT: Point-of-Care Testing
POPs: Persistent Organic Pollutants
PLGA: Poly­(lactic-*co*-glycolic acid)
ROS: Reactive Oxygen Species
SANs: Single-Atom Nanozymes
SOD: Superoxide Dismutase
SUs: Sulfonylurea Pesticides
TCh: Thiocholine
TMB: 3,3‘,5,5′-Tetramethylbenzidine
